# Gene expression‐based drug repurposing to target aging

**DOI:** 10.1111/acel.12819

**Published:** 2018-08-09

**Authors:** Handan Melike Dönertaş, Matías Fuentealba Valenzuela, Linda Partridge, Janet M. Thornton

**Affiliations:** ^1^ European Molecular Biology Laboratory, European Bioinformatics Institute Wellcome Genome Campus Hinxton Cambridge UK; ^2^ Department of Genetics, Evolution and Environment, Institute of Healthy Aging University College London London UK; ^3^ Max Planck Institute for Biology of Aging Cologne Germany

**Keywords:** aging, drug repurposing, gene expression, the Connectivity Map

## Abstract

Aging is the largest risk factor for a variety of noncommunicable diseases. Model organism studies have shown that genetic and chemical perturbations can extend both lifespan and healthspan. Aging is a complex process, with parallel and interacting mechanisms contributing to its aetiology, posing a challenge for the discovery of new pharmacological candidates to ameliorate its effects. In this study, instead of a target‐centric approach, we adopt a systems level drug repurposing methodology to discover drugs that could combat aging in human brain. Using multiple gene expression data sets from brain tissue, taken from patients of different ages, we first identified the expression changes that characterize aging. Then, we compared these changes in gene expression with drug‐perturbed expression profiles in the Connectivity Map. We thus identified 24 drugs with significantly associated changes. Some of these drugs may function as antiaging drugs by reversing the detrimental changes that occur during aging, others by mimicking the cellular defence mechanisms. The drugs that we identified included significant number of already identified prolongevity drugs, indicating that the method can discover de novo drugs that meliorate aging. The approach has the advantages that using data from human brain aging data, it focuses on processes relevant in human aging and that it is unbiased, making it possible to discover new targets for aging studies.

## 1 INTRODUCTION

Life expectancy has increased steadily in many countries worldwide. As aging is the major risk factor for multiple pathologies, including cardiovascular diseases, neurodegenerative disorders and cancer (Niccoli & Partridge, 2012), finding interventions that can increase health during aging is of importance. Lifespan of laboratory model organisms can be greatly extended by genetic and environmental interventions, which also improve health and function during aging (Clancy et al., 2001; Lucanic, Lithgow, & Alavez, 2013; Xiao et al., 2013). Many of these interventions target components of the nutrient‐sensing network and decrease the activity of IGF/insulin and/or TOR signalling (Fontana, Partridge, & Longo, 2010). Moreover, dietary restriction (DR), decreased food intake without malnutrition, can increase lifespan and further supports the importance of nutrient‐sensing pathways in aging (Fontana & Partridge, 2015).

Pharmacological intervention can also extend animal lifespan. The DrugAge database reports drug‐induced lifespan extensions up to 1.5‐fold for Caenorhabditis elegans, 1.1‐fold for *Drosophila melanogaster* and 31% for Mus musculus (Barardo et al., 2017). Some of these chemicals may mimic the effects of DR (Fontana et al., 2010). For example, resveratrol, which induces a similar gene expression profile to dietary restriction (Pearson et al., 2008), can increase lifespan of mice on a high‐calorie diet, although not in mice on a standard diet (Strong et al., 2013). Rapamycin, directly targets the mTORC1 complex, which plays a central role in nutrient‐sensing network and has an important role in lifespan extension by DR (Mair & Dillin, 2008). Rapamycin extends lifespan by affecting autophagy and the activity of the S6 kinase in flies. However, it can further extend the fly lifespan beyond the maximum achieved by DR, suggesting that different mechanisms might be involved (Bjedov et al., 2010). Nevertheless, the mechanisms of action for most of the drugs are not well known.

Several studies have taken a bioinformatics approach to discover drugs that could extend lifespan in model organisms. For instance, the Connectivity Map (CMap), a database of drug‐induced gene expression profiles, has been used to identify DR mimetics and found 11 drugs that induced expression profiles significantly similar to those induced by DR in rats and rhesus monkeys (Calvert et al., 2016). Another study generated a combined score reflecting both the aging relevance of drugs based on the GenAge database and GO annotations as well as the likely efficacy of the drugs in model organisms, using structural analyses and other criteria such as solubility (Ziehm et al., 2017). A machine learning approach has been used to identify prolongevity drugs based on the chemical descriptors of the drugs in DrugAge database and GO annotations of their targets (Barardo et al., 2017). Using DrugAge as a training set, the results reflect the known pathways in aging, and thus identified anticancer and antiinflammatory drugs, compounds related to mitochondrial process and gonadotropin‐releasing hormone antagonists. Another study took a pharmacological network approach to characterize antiaging drugs, first screening a large library of 1,280 compounds for lifespan extension in *C. elegans*. The 60 hits from the screen were used to construct a pharmacological network and clustered in certain pharmacological classes, mainly related to oxidative stress (Ye, Linton, Schork, Buck, & Petrascheck, 2014).

Whereas most studies have focussed on model organisms, one study used the known prolongevity drugs from the Geroprotectors database (Moskalev et al., 2015) and asked whether these could be functional in humans (Aliper et al., 2016). Using young and old human stem cell expression profiles, they calculate a geroprotective score based on the GeroScope algorithm, which scores drugs based on the drug targets and age‐associated expression changes in related pathways (Zhavoronkov, Buzdin, Garazha, Borisov, & Moskalev, 2014). Testing the top hits in senescent human fibroblast cultures, they suggest several geroprotectors for humans as well as showing the potential in using human gene expression data for drug studies.

Although previous studies tried to discover drugs that can affect aging, they all focus on genes or drugs related to lifespan regulation. The role of these drugs in promoting healthy aging in humans is still an open question. In this study, using gene expression data for human brain aging, we aimed to discover not only new prolongevity drugs but also those that can improve health during aging. Human brain undergoes substantial structural changes with age, including changes in brain weight, white and grey matter volumes. Accompanied by the altered intercellular communication and synaptic loss, these changes bring about cognitive decline, neurodegeneration and memory loss (Salthouse, 2009). The biological processes showing a change in expression include pathways related to synaptic and cognitive functions as well as proteostasis (Lu et al., 2004), suggesting gene expression changes in the aging brain could be used as a surrogate to find drugs to target detrimental effects.

Here, we extended the previous approaches to identification of new antiaging drugs for humans, by focusing directly on human aging data. We used a framework that does not require any prior knowledge and is thus robust to biases in the literature and databases on aging. Moreover, using human age‐series data, this methodology has the potential to discover drugs affecting both lifespan and healthspan. Through a meta‐analysis of multiple gene expression data sets, we first compiled a robust signature that characterizes aging in human brain. We then used drug‐induced RNA expression profiles deposited in CMap (Lamb, 2006) to identify a list of potential drug candidates that could influence human brain aging. We then assessed the performance of the method in relation to previous knowledge and identified novel candidate geroprotective drugs.

## 2 RESULTS

### 2.1 Analysis of age‐related changes in RNA expression in human brains

**Figure 1:**
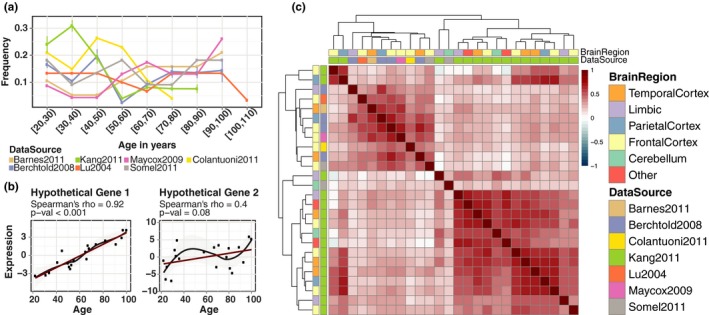
(a) Age distribution of the brains from which the data sets used in the study were derived. The error bars show the standard deviation of the sample frequency for different brain regions in data sources with multiple brain regions. (b) Hypothetical gene expression plots, demonstrating how Spearman's correlation coefficient and *p*‐value behave when the association is weak or nonmonotonic. (c) Pairwise Spearman's rank correlation coefficients across data sets. The intensity of the colours on the heatmap shows the magnitude of the correlation coefficient

We analysed data from seven, published, microarray‐based studies of age‐related changes in RNA expression (Barnes et al., 2011; Berchtold et al., 2008; Colantuoni et al., 2011; Kang et al., 2011; Lu et al., 2004; Maycox et al., 2009; Somel et al., 2010, 2011 ). The data came from 22 different brain regions, and the ages of the donors ranged from 20 to 106 years (Figure 1a and Supporting Information Figure S1a). The data for each brain region in each study were analysed separately, resulting in 26 data sets.

To characterize the association between the gene expression and age, we calculated the Spearman correlation between the expression level and age, for each gene, in each data set separately. We first calculated the number of significant changes (FDR‐corrected *p* < 0.05) in each data set (Supporting Information Figure S2). Whereas there were two data sets with a large number of significant changes, most of the data sets did not show substantial significant change. This can be explained by several factors, most importantly (a) most of the data sets had a small sample size, providing insufficient power to detect changes in most of the cases; and (b) Spearman's correlation test calculates significant monotonic changes, whereas it is likely that many of the changes are not exclusively monotonic throughout aging. Thus, we applied another approach, using the correlation coefficient to capture significant trends across data sets, instead of within a data set (see Methods). Whereas the *p*‐value is affected by the number of the samples and the strength of the monotonic relationship (Figure 1b), the sign of the correlation coefficient can be used to capture consistent trends of up‐ or downregulation once coupled with an appropriate testing scheme. This strategy requires the data sets to be concordant and reflect genuine age‐related changes. We first investigated whether this assumption was valid. To assess the concordance among data sets, we used Spearman's correlation coefficients and calculated the correlation between expression–age correlations between data sets (Figure 1c). We observed a weak correlation with a median pairwise correlation coefficient of 0.29. To calculate the significance of this correlation, we developed a stringent permutation scheme specifically designed to account for the dependence between genes as well as the data sets (see Methods for detail). We concluded that a median correlation coefficient of 0.09 would be expected by chance and that our observation (median *ρ* = 0.29), is statistically significant (*p* < 0.001). Based on these correlations, data sets clustered according to the data source rather than to the brain region. This observation is in line with the previous studies suggesting that aging‐related changes are small and heterogeneous, making them difficult to detect (Somel, Khaitovich, Bahn, Pääbo, & Lachmann, 2006). We therefore tested for significant correlations across data sets from different studies. When we excluded the correlation coefficients among the data sets generated by the same studies, we still observed a significant correlation coefficient of 0.22 (permutation test *p* < 0.001, *ρ* = −0.002 would be expected by chance), showing that we have significant correlations among different data sources as well. Using these correlations, we proceeded to compile the aging signatures, reflecting consistent trends.

### 2.2 Defining the aging signature

**Figure 2:**
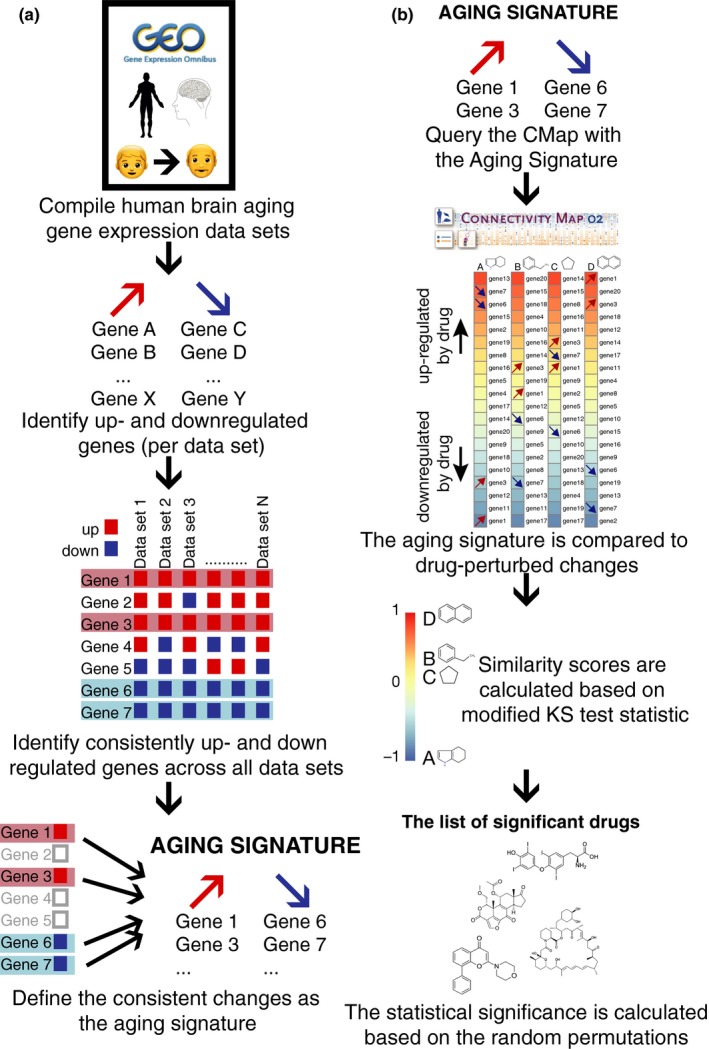
Method summary for (a) compiling the aging signature and (b) the CMap algorithm

To construct a robust aging signature, we identified the age‐related changes that were observed across all data sets, irrespective of the effect size. We thus focussed on global age‐related changes in the brain, rather than region‐specific changes, and the set of genes that showed gene expression changes in the same direction across all data sets (Figure 2a). This profile consisted of only 100 upregulated and 117 downregulated genes (Supporting Information Table S2, Figures S3 and S4), “the aging signature”.

To establish the robustness of the aging signature, we calculated the statistical significance of the number of consistent changes with the same permutation scheme used to test the correlations among data sets. This methodology randomizes the age of each individual, making it possible to test the null hypothesis where there is no association between expression and age while retaining the dependence between genes and data sets (see Methods for details). The number of consistent expression changes across brain regions was significant (*p* < 0.001; Figure S6a,b), establishing that the aging signature indeed has biological meaning.

To further test the robustness of the aging signature, we used an independent data set, consisting of gene expression in human brain generated by the GTEx Consortium (Ardlie et al., 2015), consisting of data from 99 individuals, 13 brain regions and ages between 20 and 79 (Supporting Information Figure S1a, and Table S1). These data were generated using RNA‐seq, allowing us to assess the robustness of the aging signature to different technology platforms. We used pipeline previously applied to the microarray data to calculated age‐related expression changes for each gene in each brain region separately. The pairwise correlations between the GTEx data sets were higher than with the other data set, and they tended to cluster together (Supporting Information Figure S5). We found 1,189 upregulated and 1,352 downregulated genes that showed the same direction of change across all GTEx brain regions (Supporting Information Table S2), compared with only 100 and 117 in the microarray aging signature. A likely explanation is that samples from different brain regions from the same individuals were used in GTEx, whereas the microarray aging signature combined seven independent studies and different microarray platforms. The numbers of shared expression changes based on permutations were 127 and 131.5, for down‐ and upregulated genes, suggesting a higher false positive rate in the GTEx data set. Nevertheless, the numbers of consistent up‐ and downregulated genes in the GTEx data set were also significant (*p* = 0.001; Supporting Information Figure S6c,d). The numbers of common up‐ and downregulated genes across the GTEx and microarray signatures were 50 and 48, respectively, both statistically significant (binomial test *p* < 2.2e‐16 for both), demonstrating that the aging signature was reproducible.

### 2.3 Biological processes associated with the aging signature

**Figure 3:**
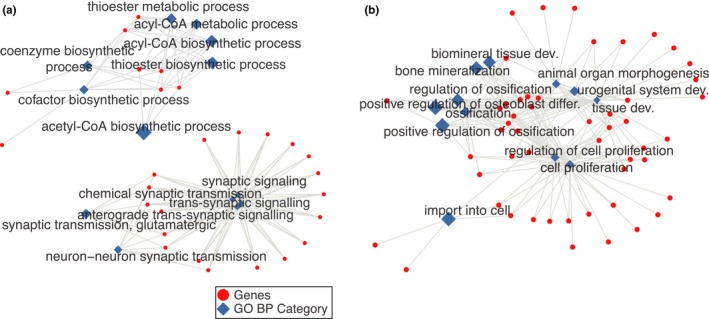
Gene Ontology Biological Process Categories significantly enriched in (a) down‐ and (b) upregulated genes in the microarray aging signature. Red circles represent the genes, and diamonds show the significantly associated GO categories, where FDR adjusted *p* < 0.05. The size of the diamonds represents the effect size (odds ratio)

We next investigated the biological processes associated with the microarray aging signature. Using the genes that were consistently expressed in all data sources as background, we did Gene Ontology enrichment tests for consistently up‐ and downregulated genes, separately (Figure 3, Supporting Information Table S3 [upregulated], Table S4 [downregulated]). Downregulated genes were enriched in synaptic functions and biosynthetic processes (FDR‐corrected *p* < 0.05), whereas differentiation and proliferation‐related categories showed enrichment for the upregulated genes (FDR‐corrected *p* < 0.05). These results are consistent with the findings of earlier brain aging transcriptome studies (Lu et al., 2004; Naumova et al., 2012; Xue et al., 2007). Oddly, ossification‐related biological processes also showed significant enrichment for the upregulated genes. However, except for one gene, these ossification‐related categories shared all genes with the more generic development‐related categories. Thus, this result could be interpreted as a general upregulation of the development‐related processes rather than ossification‐related categories.

We repeated the enrichment analysis using the GTEx aging signature and found 194 and 256 GO BP categories as significantly associated with down‐ and upregulated genes, respectively (Supporting Information Tables S7 and S8). As the number of genes in the GTEx signature is higher, we had more power to detect smaller changes and thus had a higher number of significant associations. However, the effect sizes (odds ratios) for each GO BP category calculated for microarray and the GTEx aging signature were correlated (Figure S7). Correlations between the odds ratios calculated for all of the GO categories calculated in both methods were 0.46 and 0.37, for the enrichment in the down‐ and upregulated genes, respectively. Correlations increase when we considered only the GO categories that are significantly associated with at least one of the aging signatures: 0.55 and 0.60, for the enrichment in the down‐ and upregulated genes, respectively. This further shows that the aging signatures are robust. The categories enriched in downregulated genes included biological processes related to neuronal and synaptic functions, autophagy, posttranslational modifications and translation (see Supporting Information Table S7 for the full list). Processes related to response pathways, immune response, macromolecule organization and lipid metabolism showed enrichment in upregulated genes (see Supporting Information Table S8 for the full list). Interestingly, categories related to ossification were also among the GO categories significantly associated with upregulation, based on GTEx data.

### 2.4 Mapping the aging signature onto drug‐perturbed expression profiles

The Connectivity Map is a database of drug‐perturbed gene expression profiles (Lamb, 2006). It consists of 6,100 gene expression profiles for 1,309 drug perturbation experiments performed on five different cell lines. The CMap algorithm uses a modified Kolmogorov–Smirnov test statistic to calculate the similarity of a drug‐perturbed expression profile to the gene expression profile used to query the database. A positive similarity score means that the drug‐perturbed expression profile is similar to the query, whereas a negative score indicates a negative correlation (Figure 2b). Based on the random permutations, the statistical significance of the similarity score for each drug is calculated. Thus, the *p*‐value shows the probability of finding the same association when a random signature is supplied. We queried the CMap database and identified drugs that showed significant associations in either direction with the aging signatures. To determine the robustness of this procedure, we queried the CMap data using the microarray aging signature, and the top 500 upregulated and 500 downregulated genes from the GTEx aging signature (see Methods). The correlation was significant (*r* = 0.52, *p* < 2.2e‐16; Supporting Information Figure S3a) showing that the two aging signatures produce reproducible overlaps with the CMap database. To test the reproducibility and not bias the results due to the technology used to generate the data, we preferred not to combine aging signatures but report the resulting drug hits from the two signatures separately. Nevertheless, it is noteworthy that the drug similarity scores, generated using the overlap between signatures, show significant correlation with the lists generated using both microarray and GTEx signatures (Supporting Information Figure S12).

**Figure 4:**
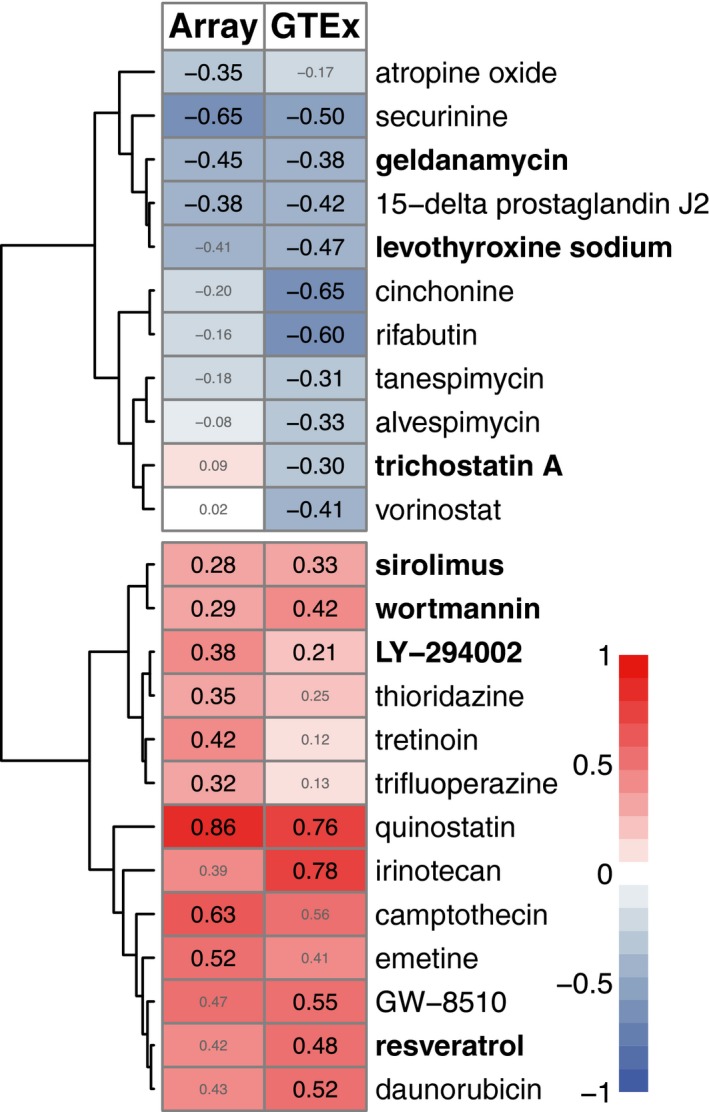
Similarity score table for the drugs having at least one significant association with the aging signatures. Each row corresponds to a drug and columns correspond to two independent aging signatures—using the microarray and the GTEx data sets. The size of score labels indicates the significance of the results (FDR‐corrected *p* < 0.05). The row labels written in bold indicates the drugs in the DrugAge database

Querying the CMap database, we identified 13 drugs significantly associated (FDR‐corrected *p* < 0.05) with the microarray aging signature (Table 1 and Figure 4). Four of these drugs were previously shown to extend lifespan in worms or flies in at least one experiment (Supporting Information Table S9). The number of prolongevity drugs rediscovered using this methodology was statistically significant (*p* = 0.004), and only one drug would be expected based on 10,000 random permutations of drugs. Repeating the same analysis with the GTEx aging signature, we identified 18 drugs, seven of which were in common with the microarray results, including the four known prolongevity drugs. In total, 24 drugs were significantly associated with at least one of the aging signatures. The correlation between the drug similarity scores for these 24 drugs calculated based on the microarray and GTEx data was 0.88 (*p* < 9.44e‐09; Supporting Information Figure S3b), indicating high concordance. As the similarity scores show high correlation, the rest of the results will be presented for the 24 drugs that are associated with at least one of the aging signatures.

**Table 1:** The drugs that are significantly associated (FDR‐corrected *p* < 0.05) with at least one of the aging signatures

Drug name	Array score	GTEx score	Target or mechanism of action
Securinine	−0.65^a^	−0.50^a^	GABRA1‐5, GABRB1‐3
**Levothyroxine sodium**	−0.41	−0.47^a^	THRA, **THRB**
Cinchonine	−0.2	−0.65^a^	**CYP2D6**
**Geldanamycin**	−0.45^a^	−0.38^a^	**HSP90AA1**
15‐delta prostaglandin J2	−0.38^a^	−0.42^a^	**PPARG**
Rifabutin	−0.16	−0.6^a^	BCL6
Atropine oxide	−0.35^a^	−0.17	–
Tanespimycin	−0.18	−0.31^a^	**HSP90AA1**
Alvespimycin	−0.08	−0.33^a^	**HSP90AA1**
Vorinostat	0.02	−0.41^a^	**HDAC1**,** HDAC2**,** HDAC3**, HDAC6
**Trichostatin A**	0.09	−0.3^a^	HDAC6, HDAC7, HDAC8
Trifluoperazine	0.32^a^	0.13	DRD2, DRD3, DRD4, HTR2A, HTR2C
Tretinoin	0.42^a^	0.12	**RARA**,** RARB**,** RARC**
**LY‐294002**	0.38^a^	0.21^a^	**PI3KCG**
Thioridazine	0.35^a^	0.25	DRD2, DRD3, DRD4, HTR2A, HTR2C
**Sirolimus**	0.28^a^	0.33^a^	**mTOR**
**Wortmannin**	0.29^a^	0.42^a^	**PI3KR1**,** PI3KCA**,** PI3KCG**
**Resveratrol**	0.42	0.48^a^	SULT1B1, YARS, LTA4H, TTR, NQO2, **PTGS2**, PTGS1, MAT2B, CSNK2A1, **CYP3A4**,** ESR1**,** PPARG**,** SIRT1**, SIRT5, CYP1A2, CYP1A1, CYP1B1, NCOA2, TNNC1
Emetine	0.52^a^	0.41	Protein synthesis inhibition
Daunorubicin	0.43	0.52^a^	**TOP2A**,** TOP2B**
GW‐8510	0.47	0.55^a^	CDK2, **CDK5**
Irinotecan	0.39	0.78^a^	**TOP1**
Camptothecin	0.63^a^	0.56	**TOP1**
Quinostatin	0.86^a^	0.76^a^	**PI3KCA**

Notes

Drug names in bold shows the drugs in DrugAge database. “Score” is the mean similarity score given in the CMap output, based on KS test.

a The similarity scores denoted with asterisk show the significant associations. The list is ordered by the mean of the similarity scores from negative to positive. Target or mechanism of action is manually curated from literature (the relevant literature is given in the Supporting Information) or extracted from CHEMBL, DrugBank and PubChem databases. The targets written in bold are found in the GenAge model organism or GenAge human databases.

Overall, the method rediscovered seven known prolongevity drugs in DrugAge database (*p* = 0.00023, based on 100,000 random permutations): resveratrol, LY‐294002, wortmannin, sirolimus (also known as rapamycin), trichostatin A, levothyroxine sodium and geldanamycin (Supporting Information Table S9).

### 2.5 Targets of the drugs

**Figure 5:**
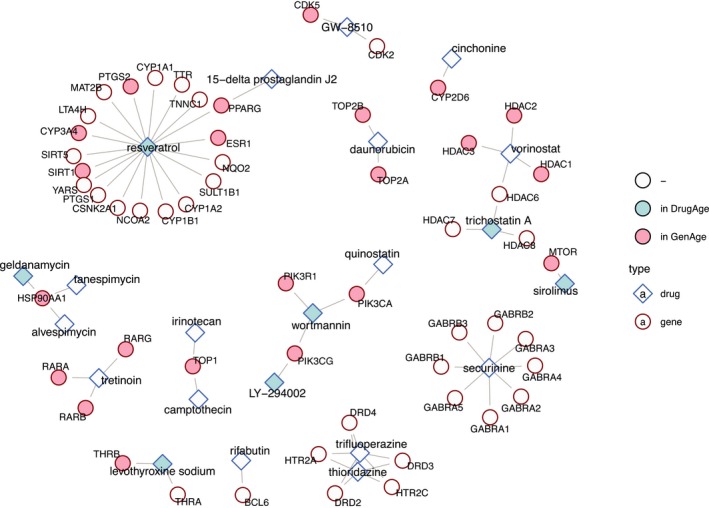
Schematic representation of the drug–target associations as a network. Blue and red nodes show drugs and targets, respectively. The drugs with a light blue background are present in DrugAge database and the targets with a pink background are in either GenAge model organism or GenAge human databases

Next, we investigated the targets of these 24 drugs, using the ChEMBL, PubChem and DrugBank databases as well as through manual curation of the literature (Table 1), and whether these targets were previously implicated in aging, using GenAge human and model organism databases (Figure 5). Except for four (rifabutin, securinine, thioridazine, trifluoperazine); all drugs or their target genes had been previously implicated in aging. Moreover, the drug–target association network showed several clusters with multiple drugs sharing the same targets: (a) Quinostatin was in the same cluster with two known prolongevity drugs, wortmannin and LY‐294002, targeting PI3 K subunits; (b) tanespimycin and alvespimycin shared the same target with another DrugAge drug, geldanamycin, targeting HSP90; (c) vorinostat shared one of its targets, HDAC6, with trichostatin A, another DrugAge drug; (d) thioridazine and trifluoperazine had dopamine and serotonin receptors as targets; and (e) irinotecan and camptothecin shared TOP1 as their target. The fact that drugs targeting the same proteins/acting through the same mechanism had similar CMap similarity scores (Figure 4) further shows that our results are biologically relevant and reflects potential mechanisms to target aging.

### 2.6 Drugs can act both by reversing aging effects and mimicking responses

The general expectation from an “omics”‐based drug repurposing study is the identification of drugs that can reverse the abnormalities detected in the disease state, that is, identification of drugs with negative similarity scores (Duran‐Frigola, Mateo, & Aloy, 2017). Following the same logic, one might expect drugs with antiaging potential to have negative scores. Interestingly, some of the known prolongevity drugs had positive similarity scores to the aging signatures, meaning that the drug‐induced profile was similar to the aging signature. A plausible explanation for this observation is that aging signatures may partly reflect cellular defence responses, helping to alleviate the damaging effects of aging.

### 2.7 Characterizing the biological functions associated with prolongevity drugs

To identify the biological processes associated with the changes that were reversed or mimicked by the prolongevity drugs, we used the drugs documented in DrugAge that were rediscovered in our analysis. We grouped the microarray aging signature into five categories, based on the expression changes in aging (up or down), and the prolongevity drug‐induced profile (up, down or inconsistent; Supporting Information Table S5). To compile the prolongevity drug profile, for each probe‐set in the microarray aging signature, we asked whether the seven DrugAge drugs induced similar changes. If the same direction of change was induced by more than half of these DrugAge drugs, then we included these changes in the prolongevity drug profile (see Methods for the details). We then analysed the biological processes associated with the genes in these categories. The number of genes is small, with no significant changes after multiple test correction. We therefore report the associations based on the highest odds ratios only. For genes downregulated in aging, the changes mimicked by the drugs were associated with autophagy and metabolic processes (Supporting Information Table S6), whereas for upregulated genes, prolongevity drugs tended to mimic the changes in protein complex/cellular complex assembly‐related functions and to reverse the changes observed in protein localization and immune‐related functions (Supporting Information Table S6). These findings are consistent with the mechanism of action for the most well‐known prolongevity drugs. For example, sirolimus (rapamycin) is an immunosuppressant approved for human use, and similar drugs can enhance the response of elderly humans to immunization against influenza (Mannick et al., 2014).

### 2.8 Similarity among significant drugs based on the expression changes at the functional level

To analyse the similarities among drugs based on expression level changes, we performed a gene‐set enrichment analysis (GSEA) for the drug‐induced expression profiles, including all genes irrespective of whether a given gene is in the aging signature (see Methods). To measure the similarity between drugs, we calculate the Spearman rank correlation coefficients between the enrichment scores and then cluster drugs based on these correlation coefficients. Notably, drugs targeting the same proteins or pathways, for example, PI3 K inhibitors LY294002, wortmannin and quinostatin, clustered together. Using this functional level approach, we grouped drugs into four groups: (a) known prolongevity drugs; (b) drugs clustering together with at least one prolongevity drug; (c) drugs which clustered together but did not cluster with any known prolongevity drugs; and (d) drugs which did not cluster with any other drugs (Supporting Information Figure S10).

### 2.9 Aging signature in other tissues

As our analysis is based on an aging signature compiled using only the brain tissue, we also explored whether this signature is representative of the other tissues. A plausible way to approach this question is repeating the same analysis using other tissues. However, it is not straightforward because (a) the number of data sets available for the other tissues limits the capacity of our approach to compile consistent signatures, increasing false positives; and (b) we find that the aging‐related changes in other tissues are not as consistent as in brain (Supporting Information Figure S11a). Thus, we choose another approach and asked whether the direction of change for the aging signature we compiled is similar to the direction of change in other tissues (Supporting Information Figure S11c). We also tested the significance of the similarity in the direction of change based on random permutations. As expected, GTEx brain data showed the highest per cent similarity to the array signature. Eight of 35 data sets showed more dissimilarity for the downregulated genes (i.e., per cent similarity was lower than 50%), whereas only two were statistically significant, namely, liver and atrial appendage. Similarly, only 6/35 data sets showed more dissimilarity for the upregulated genes, whereas none was significant. We repeated the analysis with the GTEx signature and observed similar results with only exception that there were five data sets with significant dissimilarity for the downregulated genes (Supporting Information Figure S11e). Thus, it is possible that brain signature includes some brain‐specific changes but based on significant similarity, we can say it is also representative of other tissues.

## 3 DISCUSSION

In this study, using gene expression data, we identified a set of drugs that are likely to modulate aging in the human brain. Using a meta‐analysis approach, we generated a reproducible aging signature that represents multiple brain regions and is independent of the platform used for the detection of expression. Using CMap, we identified drugs highly associated with this aging signature. Based on the DrugAge database, seven of these drugs were previously tested on model organisms and prolonged lifespan in at least one experiment. The fact that we successfully rediscovered a statistically significant number of known lifespan modulators, without using any prior drug aging information, suggests that the other drugs that we identified also have a high potential to be modulators of the aging process/lifespan. Eleven of these had targets implicated in aging, based on GenAge database (Tacutu et al., 2018). These targets include extensively studied aging‐modulators such as PI3 K subunits and histone deacetylases. We also identified a group of novel candidates that are not in aging databases, which can offer new targets and mechanisms to modulate aging. These include drugs targeting serine/threonine, muscarinic acid, and GABA(A) receptors, protein translation and BCL6 gene. Moreover, as we used human expression data, the drugs we identified may affect not only lifespan but also the healthspan by improving the cognitive functions. Indeed, some of the drugs or their targets, for example, tretinoin targeting RAR genes and GW‐8510 targeting CDK2 and CDK5, were previously linked to neuroprotective functions or neurodegenerative diseases. A literature research presented in Supporting Information provides more information on the potential mechanisms of these top drugs and their potential effect on both lifespan and healthspan in humans.

“Omics”‐based drug repurposing studies, such as the CMap, aim to identify drugs reversing the profile induced by a biological state of interest. Aging is a time‐dependent, complex phenomenon, which induces subtler changes compared to development (Dönertaş et al., 2017), or to a disease state such as Alzheimer's (Avramopoulos, Szymanski, Wang, & Bassett, 2011). The “omics” profile reflects two potentially distinct contributions: the detrimental effects which occur with age (e.g., accumulation of mutations) and the potentially beneficial responses to those changes (e.g., the immune response). As a result, CMap similarity score is not conclusive on its own. To characterize the potential effects of drugs on aging (anti‐ or proaging drugs), we use three different approaches: (a) comparison of the drug‐induced expression profiles with the known prolongevity drug profile (Supporting Information Figure S9); (b) functional analysis of the drug‐induced gene expression changes (Supporting Information Figure S10); and (c) compilation of literature on the drugs and targets (Supporting Information). On the basis of these analyses we suggest that eight of seventeen drugs (quinostatin, trifluoperazine, thioridazine, vorinostat, alvespimycin, tanespimycin, rifabutin and 15‐d prostaglandin J2), which are not in DrugAge, are likely to have positive effects, whereas topoisomerase inhibitors (camptothecin, irinotecan and daunorubicin) can be detrimental and could act as proaging drugs. Four of the remaining drugs, which are cinchonine, securinine, emetine and tretinoin, do not cluster closely with any known prolongevity drugs in Supporting Information Figure S10. Literature, however, suggests cinchonine and securinine are likely to have negative effects (see Supporting Information), whereas emetine and tretinoin could act as antiaging drugs. GW‐8510 and atropine oxide could not be classified because neither the clustering results nor literature evidence are conclusive.

It is important to note that none of the cell lines used to generate the CMap data originates from the brain. The assumption for using the CMap algorithm is that the effect we see in diverse cell lines reflects the global profile of the drug perturbation and thus should be also transferable to the brain. However, it is possible that drugs have cell or tissue‐specific effects. Even if the drugs induce the same expression changes in brain cells, an important question is: Can they cross the blood–brain barrier to target the brain? If some of these drugs have side effects on the CNS, it might be an indication that these drugs can affect the brain and can be repurposed to target brain aging. Only eight of the 24 compounds have reported side effects, and all of them have at least one reported effect on the nervous system, based on MedDRA system organ classes (Supporting Information Table S10). This implies that these drugs can affect CNS, although we do not have information on their ability to cross the barrier. The rest may or may not cross the barrier to influence the expression in the brain, but they may also improve health by targeting generic changes throughout the body. The aging signatures from brain tissue show a modest but significant similarity to expression profiles from nonbrain tissues (Supporting Information Figure S10). Thus, it is possible that we identified not only drugs specifically targeting aging in the brain but also drugs targeting aging in other tissues. It is also possible that there are drugs which can target brain aging with more potency, but we cannot identify them because we do not have drug‐induced expression profiles for brain cells. Another important technical drawback is that the data we used to generate the aging signature are bulk RNA expression data sets, where the expression profile is an average of all the cell types in the human brain. Focusing on the changes that are observed ubiquitously across all brain regions, we aimed to focus on global changes which are unlikely to be driven by cell type differences. However, future data sets generated using single‐cell expression profiling can greatly improve the understanding of both the aging process itself and how the interventions work.

To summarize, this study provides an unbiased identification of drugs that can target human brain aging. We first compiled a set of gene expression changes that can characterize human brain aging and asked whether there are drugs which alter the expression of the same genes. We identified 24 drugs, seven of which were among known prolongevity drugs. Our analysis suggests that antiaging drugs may act by mimicking the response, whereas it is also possible that they can reverse the detrimental changes in aging. On the basis of the literature research, we concluded that some of the drugs we identified can directly modulate the lifespan, whereas some are more likely to function by improving the cognitive functions and promoting the healthy aging. We are in the process of experimentally testing a group of the drugs that we have identified. We hope the information presented in this study will guide research community to further test and identify chemical modulators of the aging process in humans.

## 4 METHODS

### 4.1 Data sets

To define the gene expression changes during aging, we only included data sets with samples across different ages. In this way, we calculated the changes that occur monotonically throughout the aging process, rather than looking at differences in the young and old group. Data sets used in this study are all published data sets and include both microarray and RNA‐seq data. The preprocessing steps for each are described below.

#### 4.1.1 Microarray data sets

We used seven microarray‐based RNA expression studies with samples from 22 brain regions that are not mutually exclusive (Supporting Information Table S1). Data from different brain regions are processed and analysed separately, resulting in 26 data sets. The number of individuals in each data set ranges between 11 and 148. The total number of individuals is 304, and the total number of samples is 805 (after removing the outliers). Some studies include samples covering the whole lifespan. However, in this study, we only considered samples above 20 years of age, which corresponds to the age at first reproduction in human societies (Walker et al., 2006). Previous human brain aging studies using transcriptome data have also suggested gene expression patterns before and after the age of 20 are discontinuous (Colantuoni et al., 2011; Dönertaş et al., 2017). As we are interested in finding consistent tendencies in terms of the direction of change, which can characterize aging, we only included samples above 20 years of age. As a result, the samples included in the analysis had ages between 20 and 106. The microarray data were downloaded from NCBI GEO (Barrett et al., 2013) using the accession numbers in Supporting Information Table S1. Using “affy” (Gautier, Cope, Bolstad, & Irizarry, 2004) or “oligo” (Carvalho & Irizarry, 2010) libraries in r, RMA background correction is applied to the expression data. The data are then log2‐transformed and quantile‐normalized (using “preprocessCore” library in r). By visual inspection of the first and second principal components of the probe‐set expression levels, outliers were excluded from the further analysis (Supporting Information Table S1). The age distributions for the data sets after outlier removal are given in Supporting Information Figure S1a. Gene annotations for the probe‐sets are obtained from the Ensembl database using the “biomaRt” library (Durinck, Spellman, Birney, & Huber, 2009) in r. Because the annotations for the probe‐sets used in Kang et al. (2011) and Colantuoni et al. (2011) are not available in Ensembl, we used the GPL files deposited in GEO. If Ensembl gene IDs are not provided in the GPL files, Entrez gene IDs were extracted and converted to Ensembl Gene IDs using the “biomaRt” package. Probe‐set‐level expression information is then mapped to gene IDs. In order not to duplicate expression values, we excluded the probe‐sets corresponding to multiple genes. Expression values for the genes with multiple probe‐sets were summarized using the mean expression levels. The PCA plots for the samples using gene expression levels are given in Figure S1b.

#### 4.1.2 RNA‐seq data set

We analysed transcriptome data generated by GTEx project (v6p) (Ardlie et al., 2015). Samples are filtered based on the cause of death circumstances (4‐point Hardy Scale). Only the cases with a death circumstanceof 1 (violent and fast deaths due to an accident) and 2 (fast death of natural causes) are used for the downstream analysis and the samples with illnesses are excluded. Among all tissues, only the ones having at least 20 samples are considered. We also excluded “Cells—Transformed Fibroblasts” category to include only the samples from tissues. As a result, 35 data sets (17 major tissue type) are used for the downstream analysis, 13 of which were from the brain. The final set that we analysed includes 2,152 (623 for the brain) samples from 120 (99 for the brain) individuals. The genes with median RPKM value of 0 are also excluded from data. The RPKM values provided in the GTEx database are log2‐transformed and quantile‐normalized for the downstream analysis. Similar to the microarray data, we excluded the outliers based on the visual inspection of the first and second principal components (Supporting Information Table S1). Distribution of the ages and the PCA plots after outlier exclusion are given in Supporting Information Figure S1.

#### 4.1.3 Batch correction

In this study, each data set is analysed separately, and only the gene expression changes that are consistent across all data sets are considered for the downstream analysis. As multiple data sets are not combined, and data sets generated at different laboratories using different platforms unlikely to have the same confounders, we did not apply a correction method other than quantile normalization and outlier removal based on the PCA (using probe‐set‐level expression data for microarrays and gene‐level expression data for RNA‐seq as described above). Moreover, most of the data sets have a homogenous sample set as the number of samples is low and for the data sets with a large number of samples, we do not detect any clustering.

### 4.2 Age‐related expression changes and the aging signature

The Spearman rank correlation coefficients between age and gene expression levels are used to measure age‐related expression changes. Instead of combining the data sets, we calculated the Spearman correlation for each gene, for each data set separately. As a result, each gene had two measures to assess its age‐related expression: (a) a correlation coefficient (*ρ*), indicating the strength and the direction of change with age; and (b) a *p*‐value, showing the significance of the association. The *p*‐values are corrected for multiple testing using p.adjust function in r, with method = “FDR” argument. As the power to detect significant changes in each data set is different and the sample size is small for most of the data sets, for the downstream analysis we only used the correlation coefficients (*ρ*) and assessed the significant gene expression change tendencies that are consistent in all data sets. When a gene is upregulated by age throughout the lifespan, then it would have a positive Spearman's correlation coefficient that is close to one. In contrast, a gene would have negative correlation coefficient if it is downregulated. When the association is not strong, the magnitude of the correlation coefficient decreases, but the sign still reflects the direction of change that is observed in most of the time points. We used the sign of correlation coefficient, that is, the direction of change, to compile the set of genes that show consistent changes across all data sets. This set of genes is referred to as the “aging signature.” The aging signature, thus, does not reflect the dramatic changes in gene expression but captures consistent trends that are observed across all data sets. The statistical significance of the aging signature is calculated using a permutation scheme, testing the significance of the consistency.

### 4.3 Permutation test

We used a permutation scheme that we developed earlier (Dönertaş et al., 2017), to simulate the null hypothesis that there is no association between age and the gene expression, while retaining the dependence between genes and the data sets. Particularly, the ages of individuals in each study are permuted (randomized) 1,000 times, and if that individual donated multiple samples for different brain regions, each sample is annotated with the same age. Then, the Spearman correlation coefficient between these randomized ages and the gene expression value for all genes are calculated. In this way, we retain the dependence between genes (e.g., those regulated by the same transcription factor) and the samples (e.g., donated by the same individuals). Permutations are performed using “sample” function in base r.

Using the correlation coefficients calculated through permutations performed as explained above, we tested (a) significance of the correlations among data sets; (b) significance of the finding the same or a higher number of consistently up‐ or downregulated genes, that is, the aging signature. To test the significance of the correlations among data sets, we calculated the correlations between the expression–age correlation coefficients calculated using the permutations. We constructed the distribution for the median correlation coefficient among data sets (distribution of the 1,000 values) and calculated how many times the randomized values have higher correlation than the value we calculate using the real ages. In this way, we calculate an empirical *p*‐value. The median of the permuted values reflects the value that would be expected by chance. Similarly, to test the significance of the aging signature, we compiled permuted aging signatures, for 1,000 times, and asked how many times we have the same or higher value than the calculated number of genes in the microarray or GTEx aging signatures. In this way, we calculate the empirical p‐value and median of the number of shared tendencies based on permutations, reflecting what would be expected by chance.

### 4.4 Gene ontology enrichment

Using “topGO” and “org.Hs.eg.db” libraries in r, we performed a functional analysis of the aging signature. Using GO categories with more than 10 annotated genes, we applied an enrichment test for the Gene Ontology (GO; Ashburner et al., 2000) Biological Process (BP) categories.

### 4.5 Connectivity map analysis

A list of genes showing a consistent change in aging (the aging signature) is used to query CMap (Lamb, 2006). As CMap input requires probe‐set ids, the “biomaRt” package in r is used to convert the gene list to the probe‐set ids that are compatible with the CMap data. The probe‐sets that are in both up‐ and downregulated probe‐set lists are excluded from both lists. The final lists are used to query CMap database to associate the aging signature with the drug‐perturbed expression profiles in the database. The resulting *p*‐values are FDR‐corrected to account for multiple testing and adjusted *p* < 0.05 is used as the significance threshold.

The aging signature compiled using the GTEx data had more than 500 probe‐sets in both up and down lists. As the algorithm requires an input with <500 entries, we used the ones with the higher magnitude of expression change (median Spearman's rank correlation coefficients across 13 brain regions). To show that this does not bias the results, we repeated this step for 1,000 times by randomly selecting 500 of the probe‐sets in the GTEx aging signature. To automatize this process, we reimplemented CMap algorithm in r and calculated the drug similarity scores using the “rankMatrix.txt” data provided on the CMap website. Drug similarity scores generated using the top 500 and randomly selected 500 of the GTEx aging signature showed a significant correlation (median *ρ* = 0.81, range = 0.80, 0.82), suggesting that this approach does not bias the results.

### 4.6 Searching the drug databases for CMap drugs

Entries in CMap are composed of the drug names, which are generally the catalogue names for the drugs from chemical vendors. Similarly, DrugAge drugs also do not have an ID that is possible to map across different databases. The DrugAge database was retrieved on 11th May 2017, from the DrugAge website. To compare the drugs in CMap and the DrugAge, we first used the PubChem database (Kim et al., 2016) to make a transition across different sources. We obtained PubChem compound IDs for each drug in CMap and DrugAge using PubChem API accessed through r programming environment and “RCurl” and “jsonlite” libraries.

### 4.7 Targets of the drugs that are significantly associated with aging

We compiled the drug–target associations for the drugs significantly associated with aging mostly through literature research. For the cases where the database entries are manually curated and consistent, we used CHEMBL (Bento et al., 2014), DrugBank (Law et al., 2014) and PubChem (Kim et al., 2016). We downloaded GenAge model organism and human data sets (Tacutu et al., 2018) on 10th October 2017 using GenAge website. Using the human orthologues for the model organisms (genage_models_orthologs_export.tsv) and the human data set, we asked whether any of the drug targets were previously shown to be implicated in aging. To construct the drug–target network, we used “ggnetwork” package in r.

### 4.8 The prolongevity drug expression profile

To compile a set of gene expression changes that can be associated with the known prolongevity drug profile, we first downloaded the preprocessed data matrix with the drug‐induced expression changes (“amplitudeMatrix.txt” from CMap FTP server http://ftp.broadinstitute.org/distribution/cmap). Using this matrix, for the seven prolongevity drugs in DrugAge that are among the significant associations according to our analysis, we generated a prolongevity drug profile. We first identified the drug‐induced gene expression changes for each of these seven drugs and each of the probe‐sets that are in the microarray aging signature. For each drug–probe‐set pair, we take the direction of change that is observed in at least 60% of the experiments (using different doses or different cell lines) as the effect of that drug on the expression of that probe‐set. After deciding on the individual drug effects, we took the type of change observed in at least four of seven drugs as the prolongevity drug profile. The reason why we do not seek a perfect overlap among different drugs is to allow potentially different mechanism of actions to be included in the prolongevity drug profile. As a result, we got five categories: (a) increase in aging, increased by the drugs; (b) increase in aging, decreased by the drugs; (c) decrease in aging, increased by the drugs; (d) decrease in aging, decreased by the drugs; and (e) the ones that are not affected consistently by the drugs. The full list of genes in the first four categories is given as Supporting Information Table S5. We also asked whether any of the GO Biological Processes is enriched in any of the first four categories and thus did an enrichment analysis. We calculated the odds ratio for each GO category by keeping the type of change in aging the same. For example, we asked whether a GO category is enriched in genes that increase in aging and also increased by the drugs, compared to the genes that increase in aging but decreased by the drugs. Because the number of genes is small, it is not possible to detect significant associations after correcting for multiple testing, and thus, we only report the odds ratios for the categories (Supporting Information Table S6). We also compared the known prolongevity drug profile we compiled with the profile induced by the 24 drugs identified in the study (Supporting Information Figure S9). We calculated the percentage of probe‐sets that show the same type of change as the prolongevity drug profile. For this, we again only considered probe‐sets that show the same type of change in at least 60% of the experiments per drug.

### 4.9 Gene‐set enrichment analysis for drug‐induced changes

Using the “amplitudeMatrix.txt” downloaded from the CMap website, we determined the expression changes at the gene level for each drug. We first subset the matrix to include only the experiments for the 24 significant drugs we found. We then mapped the probe‐set ids (total number of probe‐sets = 22,283) to Entrez gene ids using the Ensembl biomaRt package in r. We map 19,222 probe‐sets to genes, excluding examples where the same probe‐set id maps to multiple genes (628 multigene probe‐set ids in total). The genes with more than one probe‐set id are represented by taking the median expression change induced for the probe‐sets (number of genes = 12,064). When the experiments for each drug are treated separately, we noticed that the results were confounded by cell line. Thus, we then summarized multiple experiments for each drug by taking the median of the change they induce. In this way, we trimmed the cell‐line‐specific effects. Then the expression changes (for 12,064 genes) for each drug (24 drugs) are rank ordered. Using clusterProfiler package and “gseKEGG” and “gseGO” functions, we performed GSEA for the gene expression changes induced by each drug separately. For the KEGG pathway analysis, we only considered the pathways with at least 50 genes (188 pathways), and for GO analysis, we only considered Biological Process categories with at least 50 and maximum of 200 genes (1589 categories).

### 4.10 Comparing brain aging signature to other tissues

We calculated the proportion of genes that show a change in the same direction with the aging signature compiled using brain data. The proportions are calculated for aging signatures compiled using the array and GTEx brain data, separately. We also analysed upregulated and downregulated genes separately to observe any differential pattern. To calculate the significance of similarity or dissimilarity, we performed 10,000 permutations as follows: (a) N number of genes, where *N* is the number of genes in a particular group (array/GTEx and up‐/downregulated), were selected randomly from a given GTEx data set; (b) the proportion of changes in a given direction is calculated; and (c) using the distribution of these proportions, we asked how many times we obtain a value as extreme as the proportion calculated for that tissue and assign empirical *p*‐value.

### 4.11 Side effects

Using compound PubChem IDs, we subset the Side Effect Resource (SIDER 4.1; Kuhn, Letunic, Jensen, & Bork, 2016), a database of adverse drugs reactions for marketed medicines. The latest version of SIDER codes the side effects using the Medical Dictionary for Regulatory Activities (MedDRA), an adverse event classification dictionary. To obtain term at the system level, we mapped the lowest level MedDRA terms in SIDER (LLT codes) to MedDRA System Organ Class terms (SOC codes) using hierarchical files downloadable from the MedDRA Web‐based browser (https://tools.meddra.org/wbb/). A total of eight drugs among the 24 had labelled side effects.

## AUTHOR CONTRIBUTIONS

H.M.D and J.M.T designed the study. H.M.D analysed the data with the help of M.F.V. H.M.D., J.M.T. and L.P. interpreted the results and wrote the manuscript. All authors read, revised and approved the final version of this manuscript.
