# Supplementary material for: Gene expression‐based drug repurposing to target aging
Source: Aging Cell. 2018 Aug 9;17(5):e12819. doi: 10.1111/acel.12819 (PMC6156541; doi:10.1111/acel.12819)
Supplement: Supplementary file 11 [file ACEL-17-e12819-s011.pdf]

SI Figures

a.

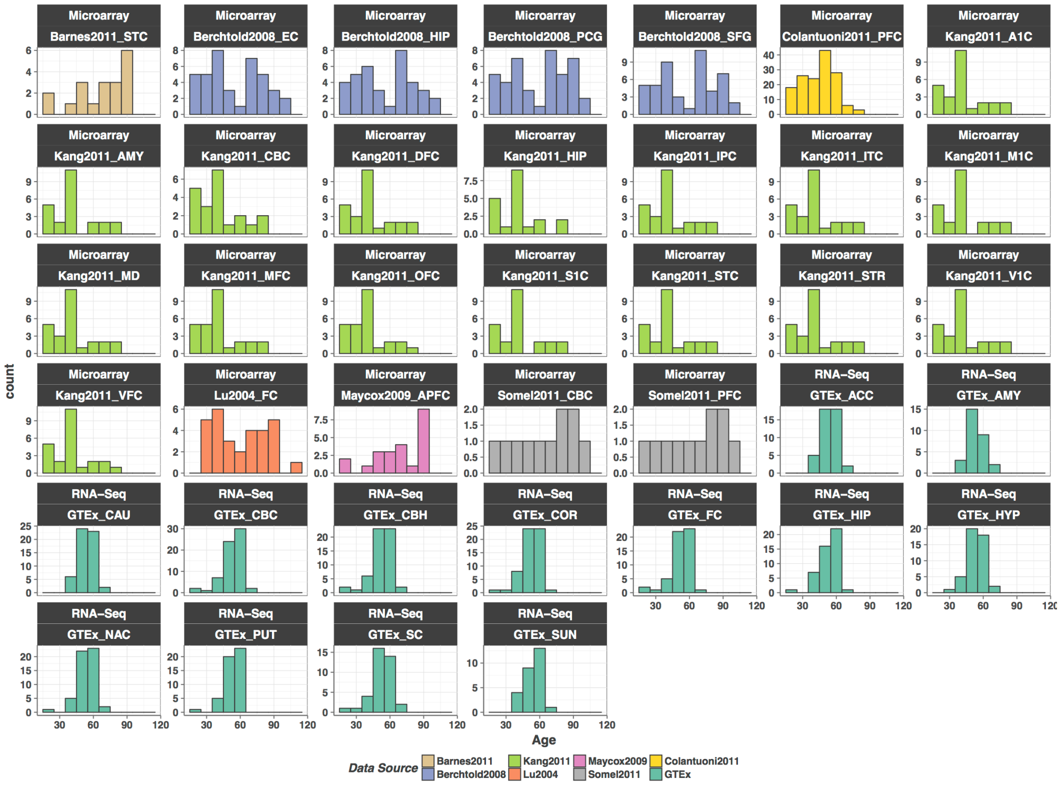

b.

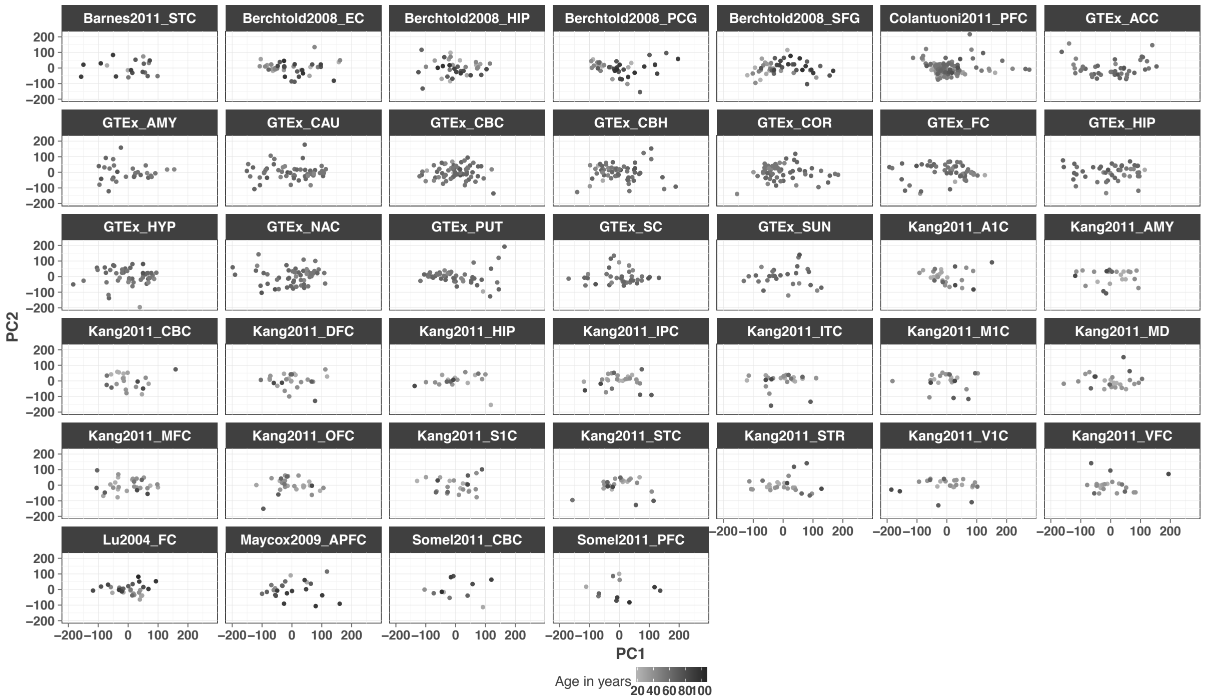

Figure S1: a) Age distribution of the sub-datasets. b) PCA plots for the sub-datasets (after outlier removal).

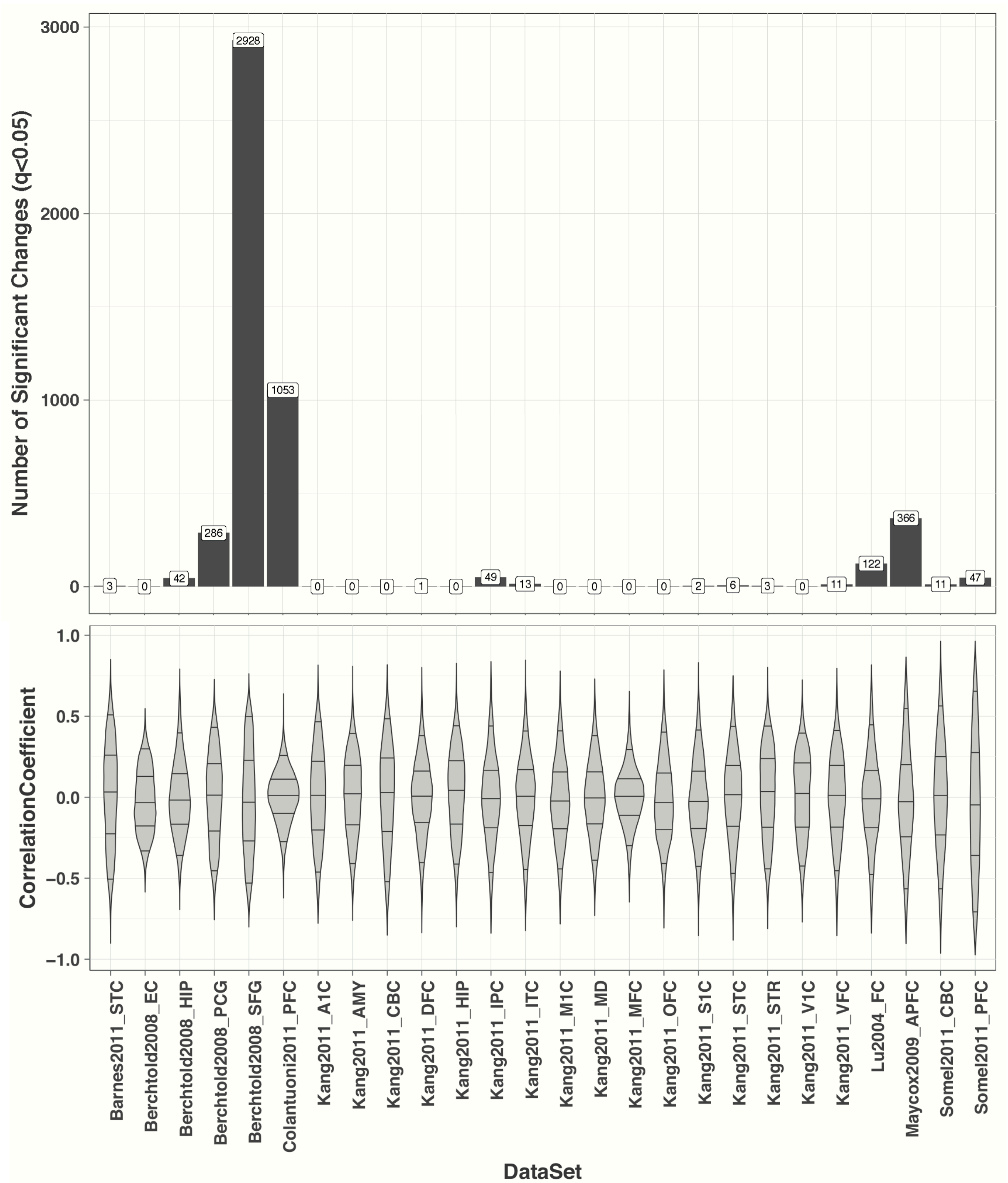

Figure S2: The number of significant changes and the distributions of Spearman's correlation coefficient between gene expression and age for each dataset.

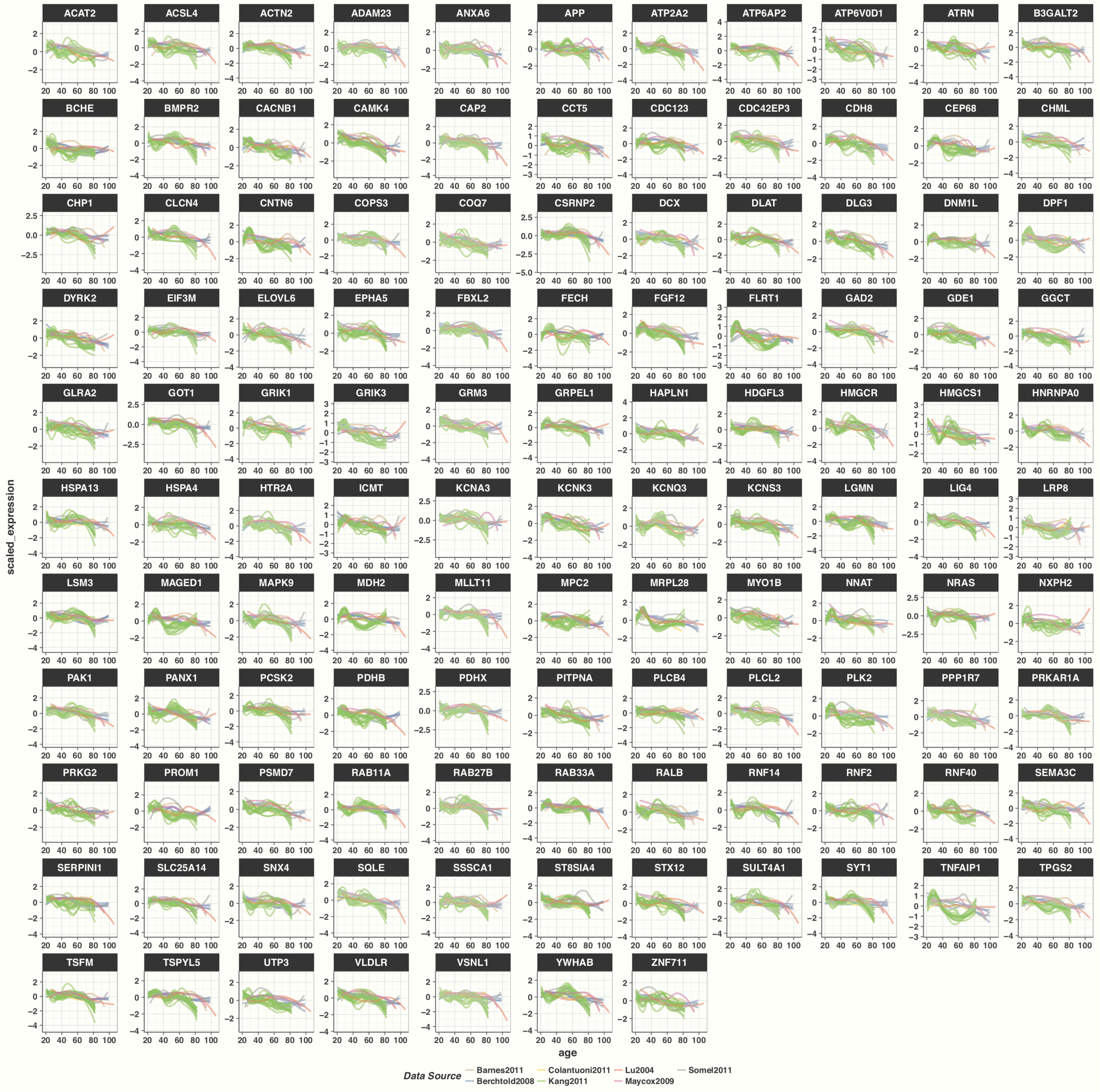

Figure S3: Scaled gene expression profile for the down-regulated genes in the microarray ageing profile. Each line shows one dataset, and the colours represent different data sources. The lines are generated using geom_smooth(method="loess") function of the ggplot2 package in R.

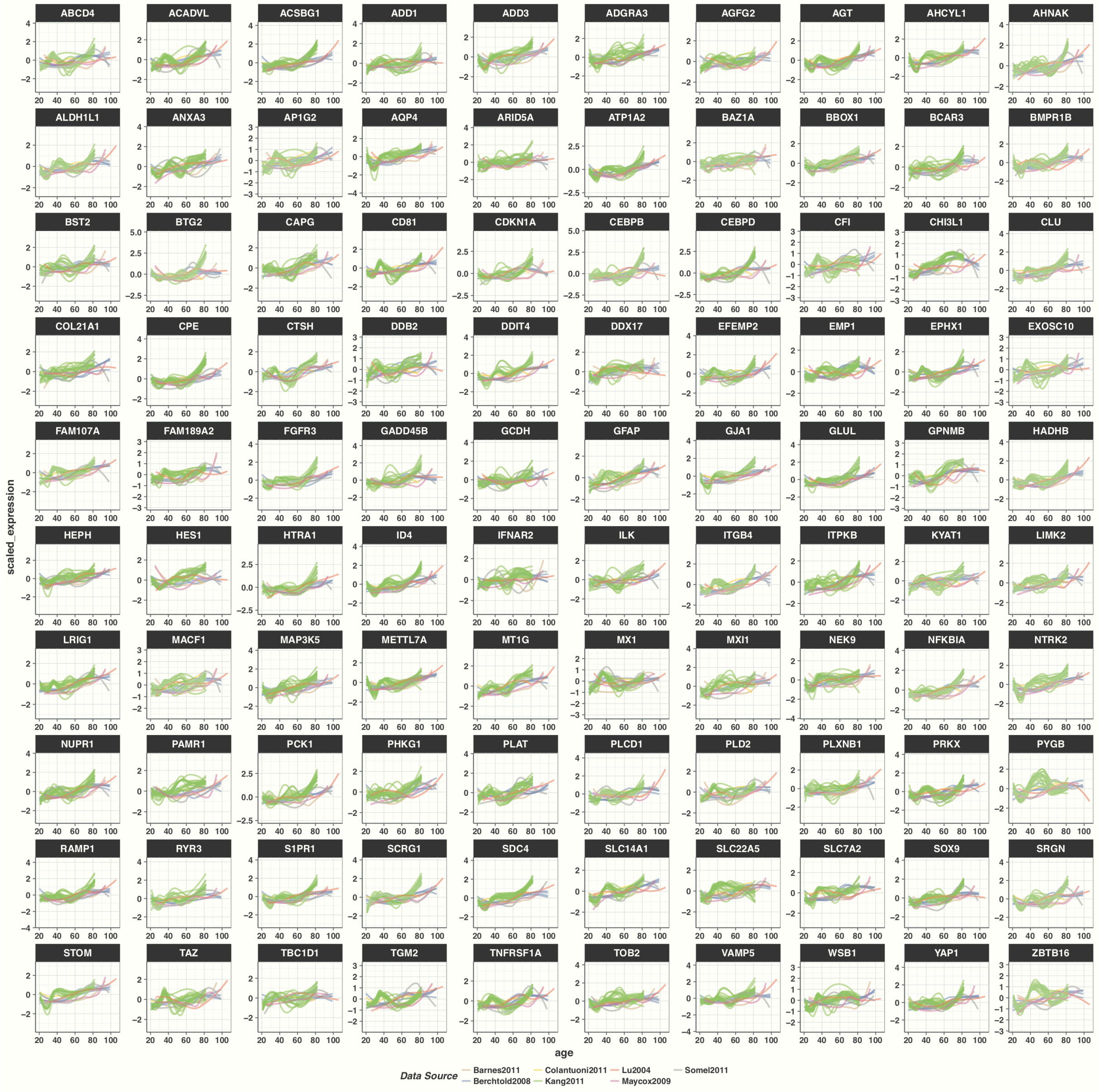

Figure S4: Scaled gene expression profile for the up-regulated genes in the microarray ageing profile. Each line shows one dataset, and the colours represent different data sources. The lines are generated using geom_smooth(method="loess") function of the ggplot2 package in R.

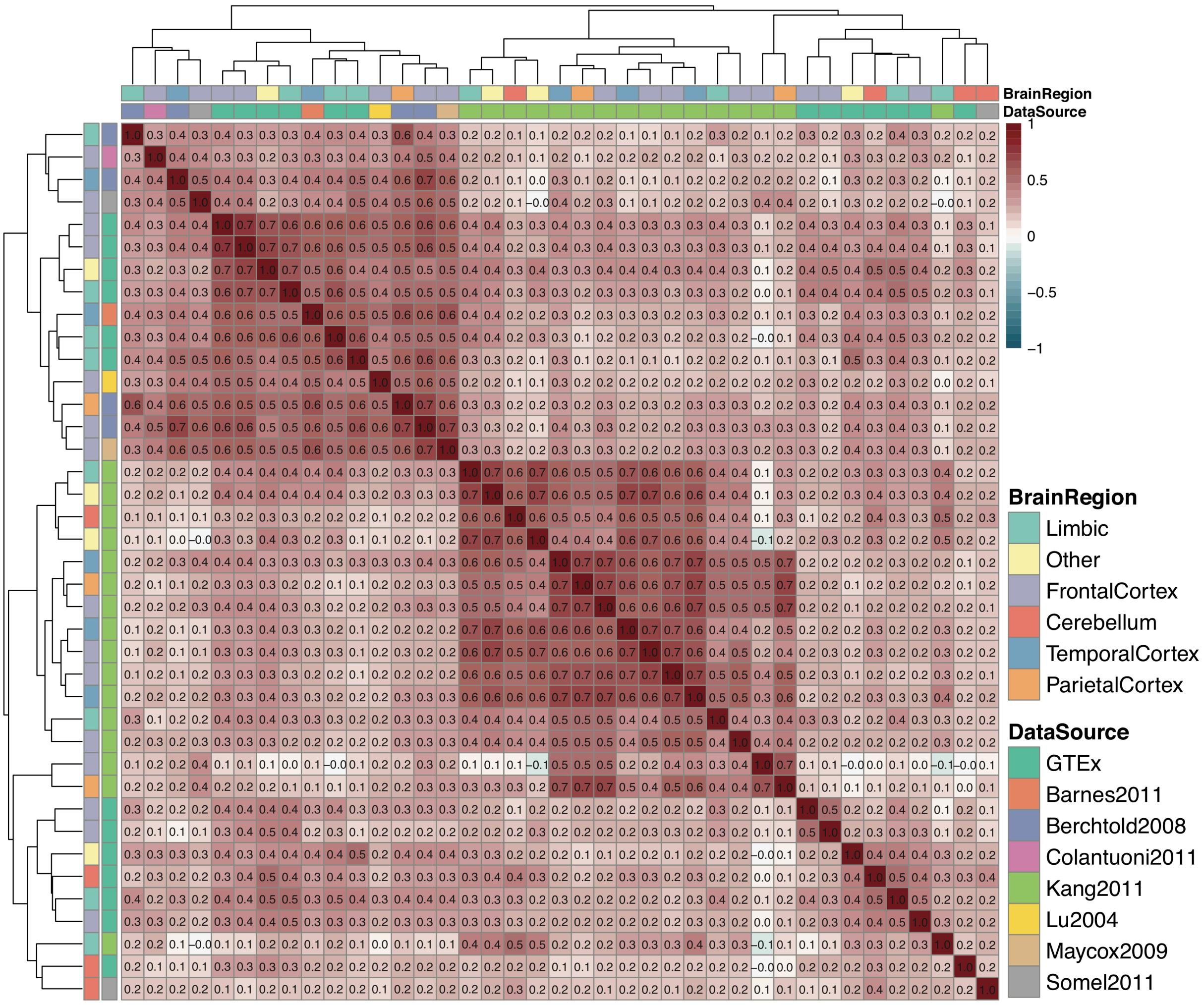

Figure S5: Pairwise Spearman's rank correlation coefficients across all datasets, including GTEx. The intensity of the colours on the heatmap shows the magnitude of the correlation coefficient.

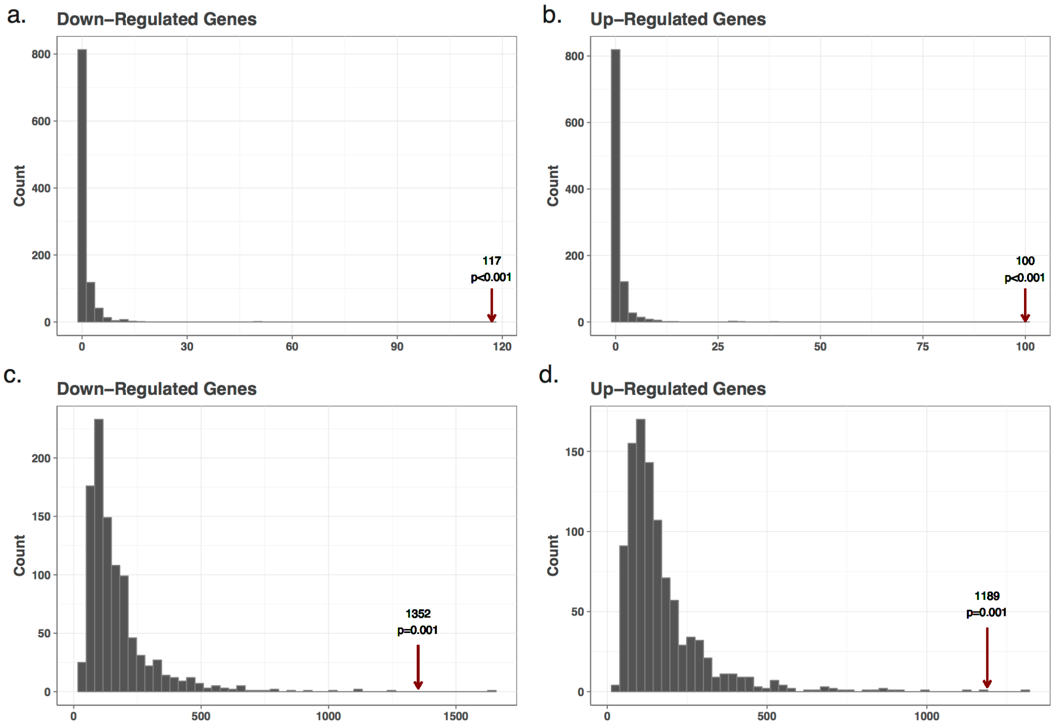

Figure S6: Distribution of the number of shared expression changes across datasets in 1000 permutations. a) Shared down-regulation across microarray datasets (expected number=0), b) shared up-regulation across microarray datasets (expected number=0), c) shared down-regulation across GTEx datasets (expected number=127), and d) shared up-regulation across GTEx datasets (expected number=131.5).

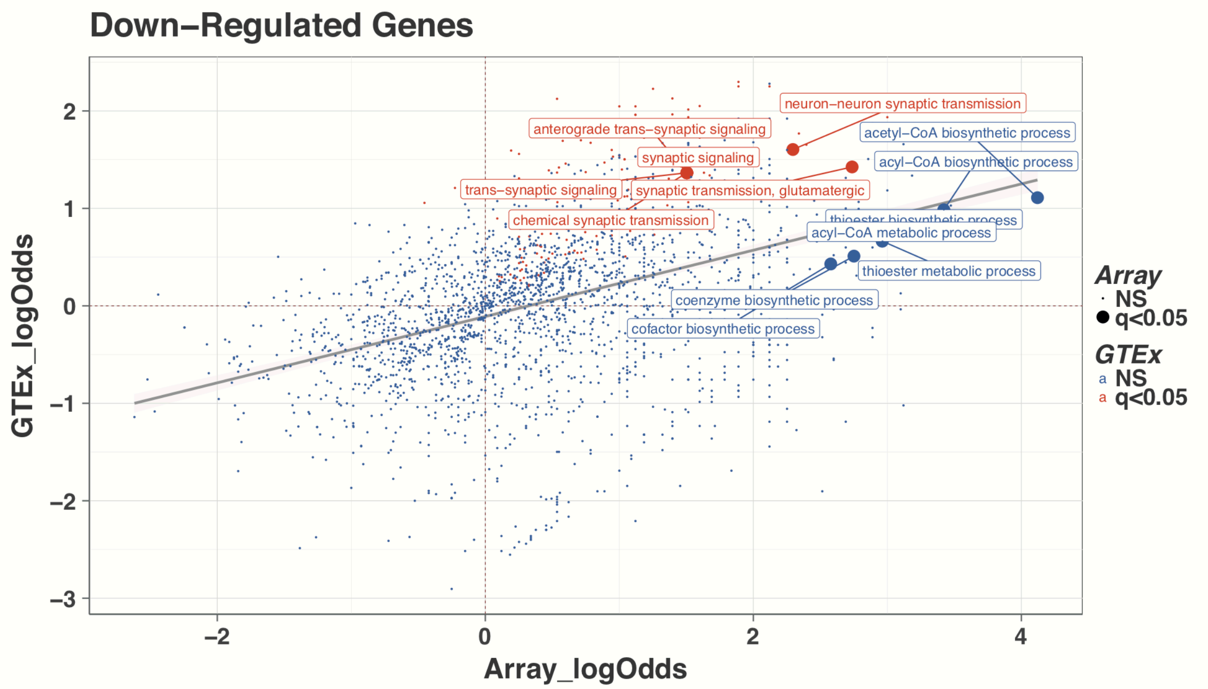

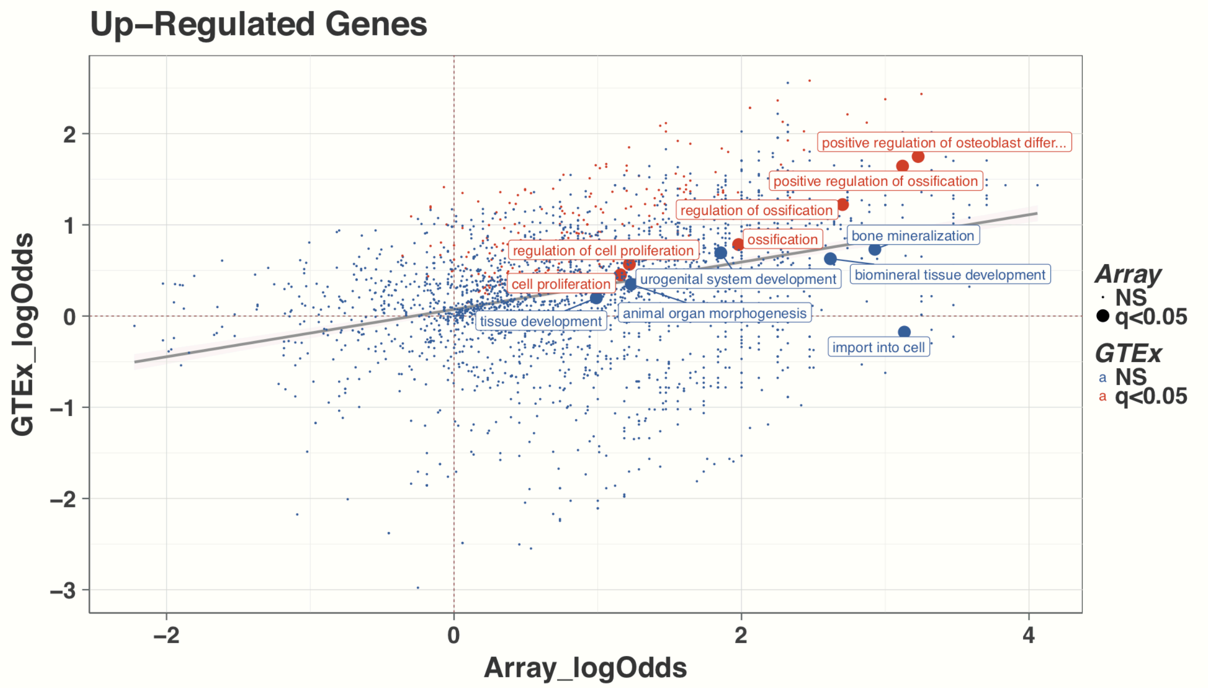

Figure S7: Scatter plot for the GO BP category log2 odds ratios calculated for the microarray and GTEx ageing signature, using Fisher's test implemented in the topGO package.

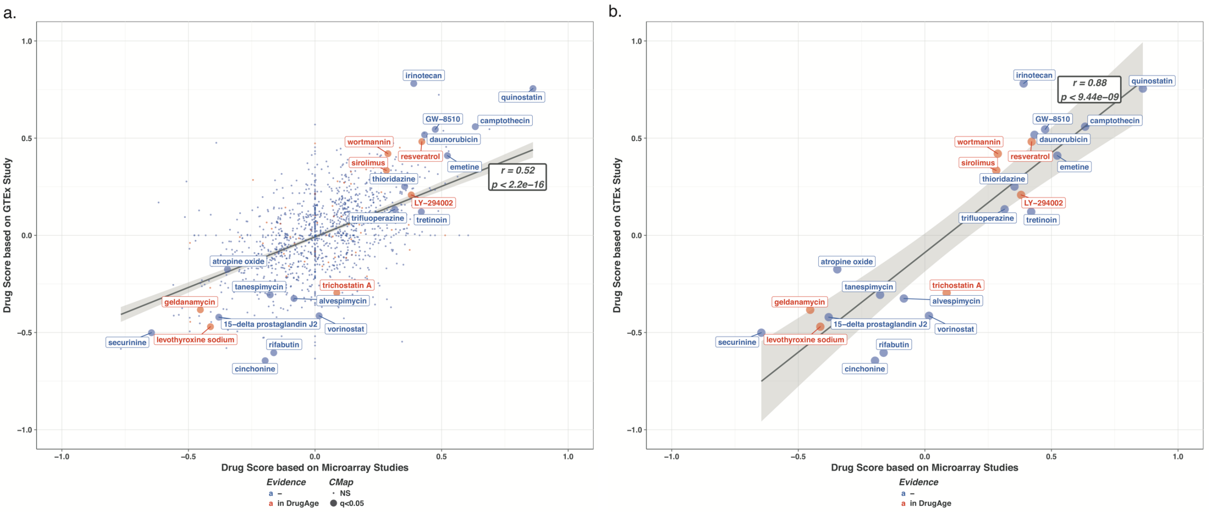

Figure S8: Scatter plot of the drug similarity scores for a) all drugs and b) only significant drugs. x-axes show the similarity score based on the microarray ageing signature, whereas y-axes show similarity score calculated using the GTEx ageing signature. The size of the data points represents the statistical significance whereas the colour shows whether a drug is previously tested on model organisms for lifespan extension (based on DrugAge database).

Characterising the potential effect of each drug on ageing

Literature search:

Eleven of the drugs have targets previously associated with ageing.
Vorinostat is a histone deacetylase (HDAC) inhibitor used for the treatment of cutaneous T-cell lymphoma ("Vorinostat," n.d.). Although not reported in the DrugAge database, vorinostat has already been tested on *Drosophila* for lifespan extension and shown to increase both mortality rate and survival when the drug is given during 'mid- to late-life' (McDonald, Maizi, & Arking, 2013). This drug had the most pro-longevity drug-like profile (Figure S9, cluster 6) based on our analysis, suggesting that the methodology, as well as the interpretation, yields biologically relevant results. ***Quinostatin***, targeting the catalytic subunit of PI3K, had the highest CMap score with percent similarity above 50% to the pro-longevity drug profile for all four categories (Figure S9). Considering that drugs targeting PI3K, such as LY-294002 and wortmannin, extend lifespan in worms and flies (Barardo et al., 2017), quinostatin is a strong anti-ageing drug candidate. ***Alvespimycin*** and ***tanespimycin*** inhibit the heat shock protein HSP90, which is also inhibited by geldanamycin. Heat shock proteins are implicated in ageing based on both experiments on worms and flies (Tacutu et al., 2017) and human expression studies. Protein aggregation and disrupted proteostasis are a hallmark of ageing (López-Otín, Blasco, Partridge, Serrano, & Kroemer, 2013). It is thus plausible that increased activity of HSP90 would reverse the effects of ageing by restoring proteostasis, although its downstream effects might result in reverse (Fuhrmann-Stroissnigg et al., 2017; McClellan et al., 2007). ***Tretinoin*** is a retinoic acid receptor (RAR) agonist widely studied for skin (Mukherjee et al., 2006) and brain ageing (Enderlin et al., 1997). RAR genes in are implicated in synaptic plasticity, learning, memory, and pathological conditions such as Alzheimer's disease (Lane & Bailey, 2005). **GW-8510** is a cyclin-dependent kinase 2 / 5 inhibitor and was suggested to be neuroprotective (Johnson et al., 2005).***15-d prostaglandin J2*** activates PPARG, which shows decreased expression with age, which can be restored by DR (Tacutu et al., 2017). ***Camptothecin*** and ***irinotecan*** both target TOP1, which alters the topological state of DNA during transcription and can inhibit Warner syndrome protein (WRN), which functions in DNA repair (Shamanna et al., 2016). These two drugs, as well as **daunorubicin** which targets TOP2A, may therefore worsen health status. ***Cinchonine,*** which targets CYP2D6 was similar to pro-longevity drug profile in terms of the genes up-regulated by the pro-longevity drugs but show opposite profile for the down-regulated genes. The functions that are associated with the genes down-regulated by the drugs are autophagy or immune function related categories. Most of the known pro-longevity drugs are suggested to function through inhibition of PI3K / mTOR pathways, favouring autophagy. It appears that cinchonine would not function in the same way. However, considering that it targets CYP2D6, which was shown to have a role in lifespan regulation in *C. elegans,* it is possible that this drug has a distinct mechanism to modulate ageing. Mann et al. previously suggested that expression of CYP2D6 increases with age in the human brain and is lower in Parkinson's disease (Mann et al., 2012). Considering that they suggest this protein might be important to inactivate neurotoxins, inhibiting this protein using cinchonine might function in the same direction and exacerbate ageing by down-regulating one of the cellular responses.

Novel candidates that are not in the GenAge or the DrugAge databases can offer new targets and mechanisms to modulate ageing.

Thioridazine and **trifluoperazine** are serotonin and dopamine receptor antagonists used for the management of psychoses, including schizophrenia. Thioridazine is withdrawn from the market due to its side effects related to cardiac arrhythmias. Ye et al. screened a library of compounds for lifespan extension in C. elegans and identified a couple of drugs targeting serotonin and dopamine receptor antagonists including thioridazine hydrochloride, which extends lifespan by 31% in C. elegans (Ye, Linton, Schork, Buck, & Petrascheck, 2014). Thus, it is likely that these drugs also have anti-ageing effects. **Emetine** is the principal alkaloid of the ipecac root. It is a eukaryotic protein translation inhibitor. A recent study investigated the effect of protein translation inhibition on cellular senescence. They suggest that cytoplasmic protein accumulation is an important cause of the cellular senescence and mild protein translation inhibition can prevent senescence induction in normal and tumour-derived human cells (Takauji et al., 2016). Although both this information and our results suggest that emetine can help alleviating the ageing, Takauji et al. did test the effect of emetine on senescence and could not detect any significant result. **Atropine oxide** is predicted to target muscarinic acetylcholine receptors (“CHEMBL2146145,” n.d.), which are suggested to be important for various brain functions as well as pathologies such as Alzheimer’s and Parkinson’s diseases (Langmead, Watson, & Reavill, 2008). The information regarding the effect of atropine oxide on different muscarinic acetylcholine receptors, however, is limited to make a conclusion whether this drug could be beneficial or damaging for the human brain ageing. **Securinine** is a GABA(A) receptor antagonist. GABA receptors are started to gain attention as potential targets for neurodegenerative diseases (Rissman, De Blas, & Armstrong, 2007). The drugs tested and shown to have an impact on cognitive abilities so far, however, are mainly GABA(A) agonists or GABA(B) antagonists (Li et al., 2016). Considering that GABA(A) subunit expression levels show a decrease with age (TableS2), and securinine is an antagonist, it is possible that it acts in the same direction as ageing and exacerbates it. **Rifabutin** shows high similarity to the pro-longevity drug profile and clusters together with levothyroxine sodium and geldanamycin, which are known pro-longevity drugs (Figure S9, cluster 2). Rifabutin is an antibiotic but it is reported to also target BCL6 (Evans et al., 2014). BCL6 gene is not in GenAge databases, however, there are studies linking this gene to ageing using human gene expression data (Glass et al., 2013) and through its role in cell proliferation and senescence, regulated by miR-127 (Chen, Wang, Guo, Xie, & Cong, 2013). Thus, it is possible that rifabutin helps to reduce the effect of damaging changes induced by ageing, through targeting BCL6.

Comparing the drug-induced expression profiles with the known pro-longevity drug profile

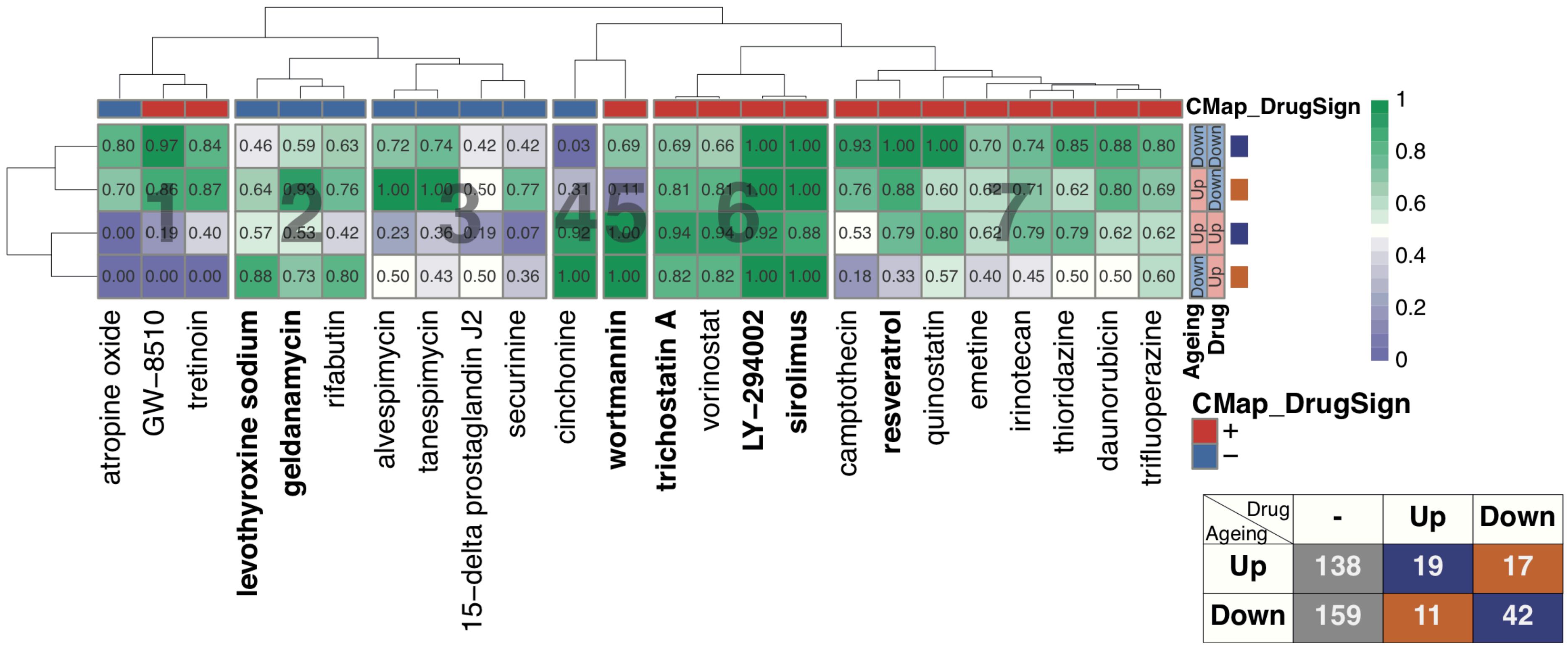

Drug / Ageing	-	Up	Down
**Up**	138	19	17
**Down**	159	11	42

Figure S9: Heatmap showing the percent similarity of each drug to the compiled pro-longevity drug profile. The numbers 1-7 show the cluster number based on the hierarchical clustering of the drugs based on the similarities. The annotation rows show the sign of similarity score based on the CMap analysis. The column labels written in bold indicates the drugs in the DrugAge database. Annotation columns show the up- or down-regulation of each category in ageing and pro-longevity drug profile. The small table shows the number of probe-sets in each category we defined to reflect pro-longevity drug profile

For each category (up or down in both ageing and the pro-longevity drug profile), we calculated the percent similarity between each drug we identified and the pro-longevity drug profile. Particularly, we calculated the percentage of the consistent expression changes induced by each drug that are in the same direction as the pro-longevity drug profile (see Methods). We generated hierarchical clustering of the drugs based on the percent similarities (Figure S9). Most of the drugs showed a similar profile for the probe-sets down-regulated by the pro-longevity drugs (except for cinchonine). The drugs with negative and positive CMap scores clustered separately, but this separation is mainly driven by just a subset of the genes – the ones up-regulated by the pro-longevity drugs. Thus, it is still possible that drugs with both negative and positive CMap scores can alleviate as well as worsen ageing, through different mechanisms of action. Overall, we divided the hierarchical tree into seven clusters to explain the trends in each group. ***Cluster 1*** had three drugs; atropine oxide, GW-8510 and tretinoin. These drugs showed a strong similarity for the probe-sets down-regulated by the pro-longevity drugs, however, they also down-regulate the probe-sets up-regulated by the pro-longevity drugs. ***Cluster 2***, which included two DrugAge drugs; levothyroxine sodium and geldanamycin, as well as a novel candidate; rifabutin, showed relatively lower, but above 50% similarity, for the probe-sets down-regulated according to the pro-longevity drug profile. These drugs were also similar to the pro-longevity drug profile in terms of the up-regulated probe-sets. ***Cluster 3*** was similar to cluster 1 but the dissimilarity in terms of the up-regulated probe-sets was less pronounced. ***Cluster 4*** consisted of only one drug showing a quite distinct profile compared to the other drugs; cinchonine. It was similar to the pro-longevity drugs in terms of the up-regulated probe-sets but it showed an opposite profile for the down-regulated probe-sets. ***Cluster 5*** was also a one-drug cluster with wortmannin, which is a DrugAge drug. Except the probe-sets up-regulated in ageing and down-regulated by the drugs, wortmannin showed similar profile to the pro-longevity drug profile. ***Cluster 6*** was the group with known pro-longevity drugs except for vorinostat. As expected, the percent similarities were the highest in this cluster. ***Cluster 7*** also showed quite a remarkable resemblance to the pro-longevity drug profile, except for the probe-sets that were down-regulated in ageing but reversed by the pro-longevity drugs, which were neither similar nor dissimilar having 50% similarity.

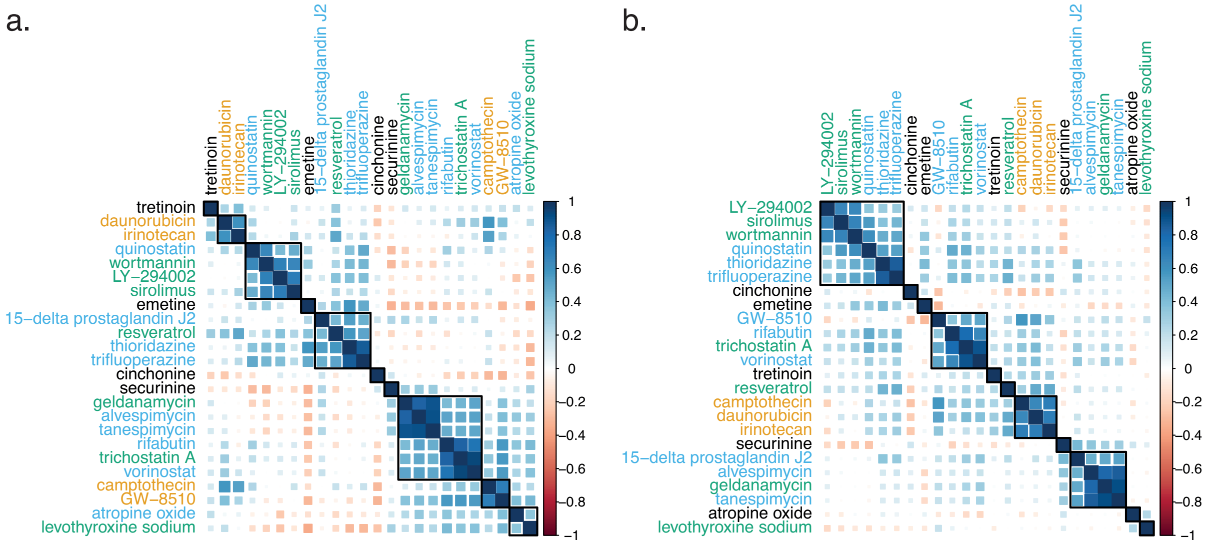

c.

	FIGS9	GO	KEGG	LITERATURE
CINCHONINE	NC	NC	NC	-
SECURININE	NC	NC	NC	-
CAMPTOTHECIN	NC	NC2	NC2	-
DAUNORUBICIN	NC	NC2	NC2	-
IRINOTECAN	NC	NC2	NC2	-
QUINOSTATIN	+	+	+	+
TRIFLUOPERAZINE	+	+	+	+
VORINOSTAT	+	+	+	+
15D-PROSTOGLANDIN J2	NC	+	+	+
ALVESPIMYCIN	NC	+	+	+
RIFABUTIN	NC	+	+	+
TANESPIMYCIN	NC	+	+	+
THIORIDAZINE	NC	+	+	+
EMETINE	NC	NC	NC	+
TRETINOIN	NC	NC	NC	+
GW-8510	NC	+	NC2	+
ATROPINE OXIDE	NC	NC	+	NC

Figure S10: a-b) Correlation matrices showing drug-drug similarities calculated using pairwise Spearman's correlation coefficients between normalized enrichment scores for a) KEGG pathways and b) GO Biological Process categories. Drugs are clustered using hierarchical clustering. The rectangles are drawn by cutting the hierarchical tree (the number of clusters is decided based on the visual inspection of the heatmap). Labels written in green are the known pro-longevity drugs based on the DrugAge database, in blue are the drugs in the same cluster with at least one pro-longevity drug, in black are the ones which did not cluster with anything and in orange are the ones that did not cluster any pro-longevity drug but are similar to each other. c) Summary matrix showing the outcome of each approach used based on Figure S9, KEGG and GO GSEA results from panel (a) and (b) and literature search given at the beginning of this section. The colour code is the same as panel (a) and (b), and dark orange shows likely negative effects, thus pro-ageing drugs.

Brain Ageing Signature in Other Tissues

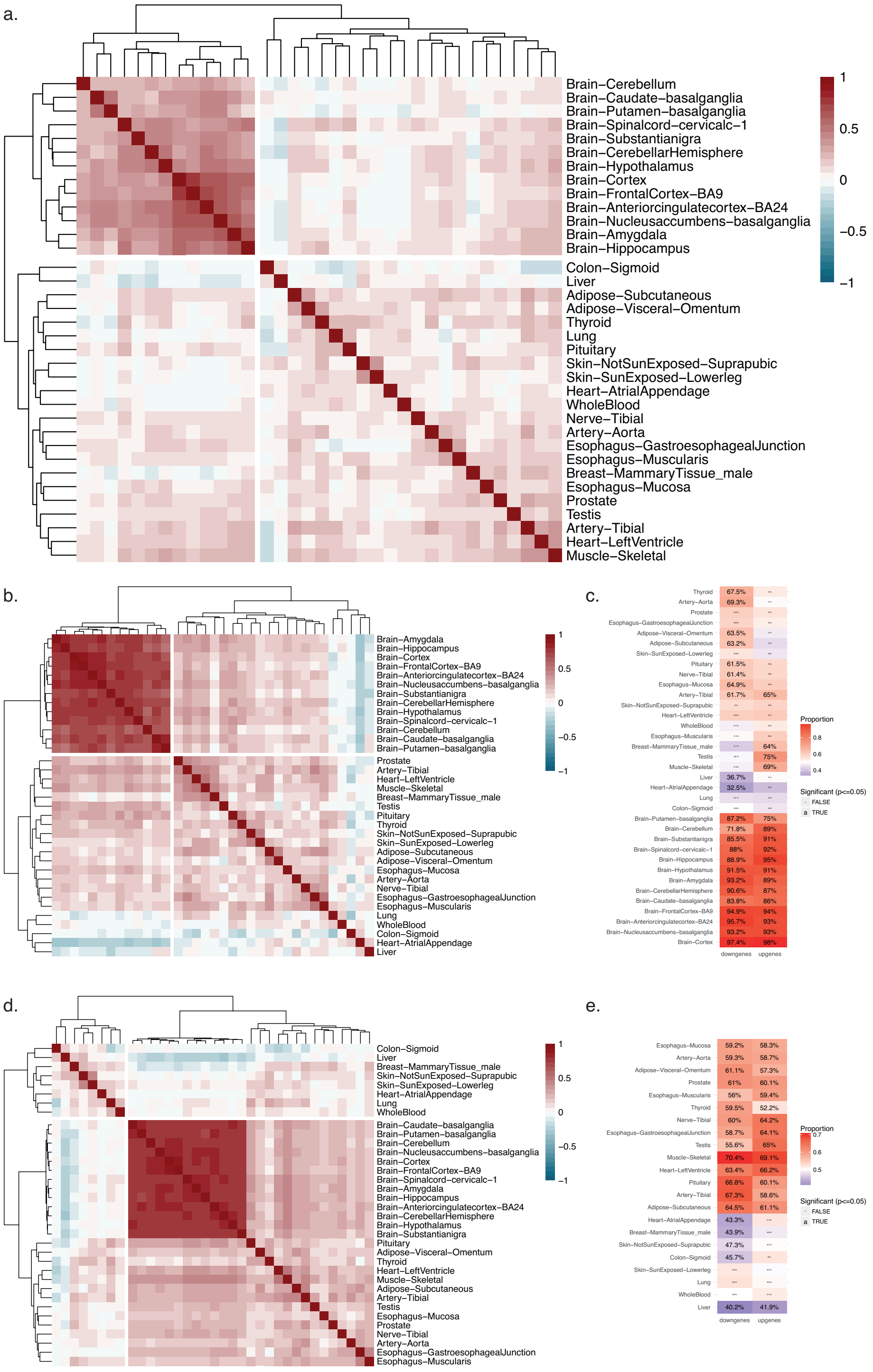

Figure S11: a) Heatmap showing the pairwise correlation coefficients among GTEx datasets, corresponding 17 major and 35 minor tissue types. The intensity of the colours on the heatmap shows the magnitude of the correlation coefficient. b and d) The same as (a) but using only the genes in brain ageing signature compiled using microarray (a) and GTEx brain dataset (b). c)Heatmap showing the proportion of the changes in the same direction with ageing signature compiled using microarray signature. The colour shows the similarity (red), and dissimilarity (blue) based on the majority of change. The labels show the proportion of the similar type of change, where the size of the labels shows the statistical significance. e) The same as (c) but for GTEx ageing signature compiled using only the brain data.

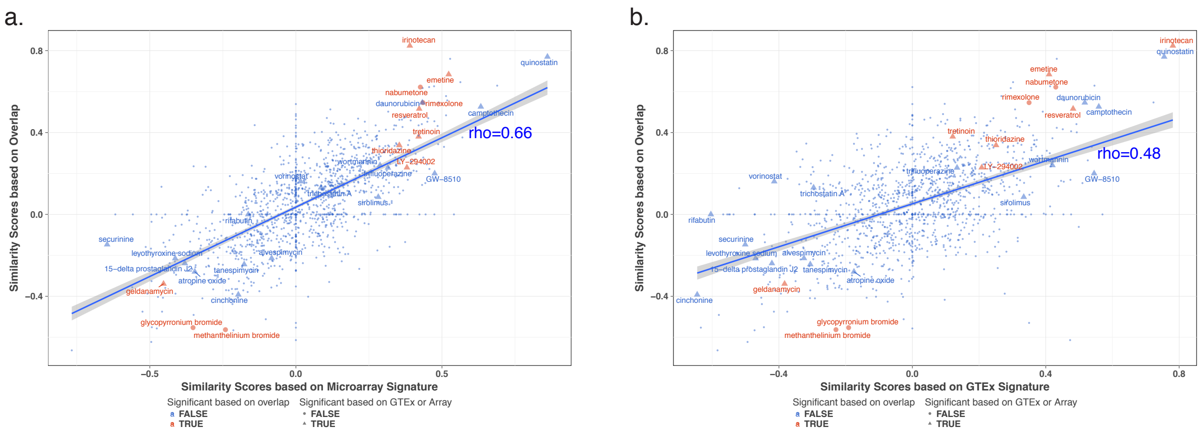

Figure S12: Scatter plot of the drug similarity scores based on the overlap of the signatures (50 up and 48 down regulated genes) and a) microarray signature and b) GTEx signature. The size of the data points represents the statistical significance whereas the colour shows whether the drug is significant based on the overlap of the signatures. The shape represents whether the drug is one of the 24 hits reported in the main text.

Table 1 with the references for drug-target associations:

Table 1: The drugs that are significantly associated (FDR corrected p<0.05) with at least one of the ageing signatures. Drug names in bold shows the drugs in DrugAge database. 'Score' is the mean similarity score given in the CMap output, based on KS test. The similarity scores denoted with (*) show the significant associations. The list is ordered by the mean of the similarity scores from negative to positive. Target or mechanism of action is manually curated from literature (the relevant literature is cited in the table) or extracted from CHEMBL, DrugBank, and PubChem databases. The targets written in bold are found in the GenAge model organism human databases. Cluster column refers to the clusters in Figure S9.

DRUG NAME	ARRAY SCORE	GTEX SCORE	TARGET OR MECHANISM OF ACTION	CLUSTER
Securinine	-0.65 (*)	-0.50 (*)	GABRA1-5, GABRB1-3 (Beutler et al., 1985)	3
**Levothyroxine sodium**	-0.41	-0.47 (*)	THRA, **THRB**	2
Cinchonine	-0.2	-0.65 (*)	**CYP2D6** (Strobl, von Kruedener, Stockigt, Guengerich, & Wolff, 1993)	4
**Geldanamycin**	-0.45 (*)	-0.38 (*)	**HSP90AA1** (Stebbins et al., 1997)	2
15-delta prostaglandin J2	-0.38 (*)	-0.42 (*)	**PPARG** (Zhao, Zhang, Strong, Grotta, & Aronowski, 2006)	3
Rifabutin	-0.16	-0.6 (*)	BCL6 (Evans et al., 2014)	2
Atropine oxide	-0.35 (*)	-0.17	-	1
Tanespimycin	-0.18	-0.31 (*)	**HSP90AA1**	3
Alvespimycin	-0.08	-0.33 (*)	**HSP90AA1** (Jez, Chen, Rastelli, Stroud, & Santi, 2003)	3
Vorinostat	0.02	-0.41 (*)	**HDAC1**, **HDAC2**, **HDAC3**, HDAC6	6
**Trichostatin A**	0.09	-0.3 (*)	HDAC6, HDAC7, HDAC8 (Decroos et al., 2014, 2015; Dowling, Gantt, Gattis, Fierke, & Christianson, 2008; Finnin et al., 1999; Hai & Christianson, 2016; Miyake et al., 2016; Schuetz et al., 2008; Somoza et al., 2004)	6
Trifluoperazine	0.32 (*)	0.13	DRD2, DRD3, DRD4, HTR2A, HTR2C	7
Tretinoin	0.42 (*)	0.12	**RARA**, **RARB**, **RARC**	1
**LY-294002**	0.38 (*)	0.21 (*)	**PI3KCG** (Walker et al., 2000)	6
Thioridazine	0.35 (*)	0.25	DRD2, DRD3, DRD4, HTR2A, HTR2C	7
**Sirolimus**	0.28 (*)	0.33 (*)	**mTOR**	6
**Wortmannin**	0.29 (*)	0.42 (*)	**PI3KR1**, **PI3KCA**, **PI3KCG**	5
**Resveratrol**	0.42	0.48 (*)	SULT1B1, YARS, LTA4H, TTR, NQO2, **PTGS2**, PTGS1, MAT2B, CSNK2A1, **CYP3A4**, **ESR1**, **PPARG**, **SIRT1**, SIRT5, CYP1A2, CYP1A1, CYP1B1, NCOA2, TNNC1	7
Emetine	0.52 (*)	0.41	Protein Synthesis Inhibition	7
Daunorubicin	0.43	0.52 (*)	**TOP2A**, **TOP2B** (Aubel-Sadron & Londos-Gagliardi, 1984; Zunino & Capranico, 1990)	7
GW-8510	0.47	0.55 (*)	CDK2, **CDK5** (Johnson et al., 2005)	1
Irinotecan	0.39	0.78 (*)	**TOP1**	7
Camptothecin	0.63 (*)	0.56	**TOP1** (Staker et al., 2005)	7
Quinostatin	0.86 (*)	0.76 (*)	**PI3KCA** (Yang, Shamji, Matchacheep, & Schreiber, 2007)	7

References

Aubel-Sadron, G., & Londos-Gagliardi, D. (1984). Daunorubicin and doxorubicin, anthracycline antibiotics, a physicochemical and biological review. *Biochimie*, *66*(5), 333–352. http://doi.org/10.1016/0300-9084(84)90018-X

Barardo, D., Thornton, D., Thoppil, H., Walsh, M., Sharifi, S., Ferreira, S., … de Magalh??es, J. P. (2017). The DrugAge database of aging-related drugs. *Aging Cell*. http://doi.org/10.1111/acel.12585

Beutler, J. A., Karbon, E. W., Brubaker, A. N., Malik, R., Curtis, D. R., & Enna, S. J. (1985). Securinine alkaloids: A new class of GABA receptor antagonist. *Brain Research*, *330*(1), 135–140. http://doi.org/10.1016/0006-8993(85)90014-9

CHEMBL2146145. (n.d.). Retrieved October 31, 2017, from https://www.ebi.ac.uk/chembldb/compound/inspect/CHEMBL2146145

Chen, J., Wang, M., Guo, M., Xie, Y., & Cong, Y. S. (2013). miR-127 regulates cell proliferation and senescence by targeting BCL6. *PLoS ONE*, *8*(11), e80266. http://doi.org/10.1371/journal.pone.0080266

Decroos, C., Bowman, C. M., Moser, J. A. S., Christianson, K. E., Deardorff, M. A., & Christianson, D. W. (2014). Compromised structure and function of HDAC8 mutants identified in Cornelia de Lange Syndrome spectrum disorders. *ACS Chemical Biology*, *9*(9), 2157–2164. http://doi.org/10.1021/cb5003762

Decroos, C., Christianson, N. H., Gullett, L. E., Bowman, C. M., Christianson, K. E., Deardorff, M. A., & Christianson, D. W. (2015). Biochemical and Structural Characterization of HDAC8 Mutants Associated with Cornelia de Lange Syndrome Spectrum Disorders. *Biochemistry*, *54*(42), 6501–6513. http://doi.org/10.1021/acs.biochem.5b00881

Dowling, D. P., Gantt, S. L., Gattis, S. G., Fierke, C. A., & Christianson, D. W. (2008). Structural studies of human histone deacetylase 8 and its site-specific variants complexed with substrate and inhibitors. *Biochemistry*, *47*(51), 13554–13563. http://doi.org/10.1021/bi801610c

Enderlin, V., Alfos, S., Pallet, V., Garcin, H., Azaïs-Braesco, V., Jaffard, R., & Higueret, P. (1997). Aging decreases the abundance of retinoic acid (RAR) and triiodothyronine (TR) nuclear receptor mRNA in rat brain: Effect of the administration of retinoids. *FEBS Letters*, *412*(3), 629–632. http://doi.org/10.1016/S0014-5793(97)00845-4

Evans, S. E., Goult, B. T., Fairall, L., Jamieson, A. G., Ko Ferrigno, P., Ford, R., … Wagner, S. D. (2014). The ansamycin antibiotic, rifamycin SV, inhibits BCL6 transcriptional repression and forms a complex with the BCL6-BTB/POZ domain. *PLoS ONE*, *9*(3), e90889. http://doi.org/10.1371/journal.pone.0090889

Finnin, M. S., Donigian, J. R., Cohen, A., Richon, V. M., Rifkind, R. A., Marks, P. A., … Pavletich, N. P. (1999). Structures of a histone deacetylase homologue bound to the TSA and SAHA inhibitors. *Nature*, *401*(6749), 188–193. http://doi.org/10.1038/43710

Fuhrmann-Stroissnigg, H., Ling, Y. Y., Zhao, J., McGowan, S. J., Zhu, Y., Brooks, R. W., … Robbins, P. D. (2017). Identification of HSP90 inhibitors as a novel class of senolytics. *Nature Communications*, *8*(1), 422. http://doi.org/10.1038/s41467-017-00314-z

Glass, D., Viñuela, A., Davies, M. N., Ramasamy, A., Parts, L., Knowles, D., … Spector, T. D. (2013). Gene expression changes with age in skin, adipose tissue, blood and brain. *Genome Biology*, *14*(7), R75. http://doi.org/10.1186/gb-2013-14-7-r75

Hai, Y., & Christianson, D. W. (2016). Histone deacetylase 6 structure and molecular basis of catalysis and inhibition. *Nature Chemical Biology*, *12*(9), 741–747. http://doi.org/10.1038/nchembio.2134

Jez, J. M., Chen, J. C.-H., Rastelli, G., Stroud, R. M., & Santi, D. V. (2003). Crystal Structure and Molecular Modeling of 17-DMAG in Complex with Human Hsp90. *Chemistry & Biology*, *10*(4), 361–368. http://doi.org/10.1016/S1074-5521(03)00075-9

Johnson, K., Liu, L., Majdzadeh, N., Chavez, C., Chin, P. C., Morrison, B., … D'Mello, S. R. (2005). Inhibition of neuronal apoptosis by the cyclin-dependent kinase inhibitor GW8510: Identification of 3′ substituted indolones as a scaffold for the development of neuroprotective drugs. *Journal of Neurochemistry*, *93*(3), 538–548. http://doi.org/10.1111/j.1471-4159.2004.03004.x

Lane, M. A., & Bailey, S. J. (2005, March). Role of retinoid signalling in the adult brain. *Progress in Neurobiology*. http://doi.org/10.1016/j.pneurobio.2005.03.002

Langmead, C. J., Watson, J., & Reavill, C. (2008). Muscarinic acetylcholine receptors as CNS drug targets. *Pharmacology & Therapeutics*, *117*(2), 232–243. http://doi.org/10.1016/j.pharmthera.2007.09.009

Li, Y., Sun, H., Chen, Z., Xu, H., Bu, G., & Zheng, H. (2016, February 23). Implications of GABAergic neurotransmission in Alzheimer's disease. *Frontiers in Aging Neuroscience*. Frontiers.

http://doi.org/10.3389/fnagi.2016.00031
López-Otín, C., Blasco, M. A., Partridge, L., Serrano, M., & Kroemer, G. (2013). The hallmarks of aging. *Cell*, *153*(6), 1194–1217. http://doi.org/10.1016/j.cell.2013.05.039
Mann, A., Miksys, S. L., Gaedigk, A., Kish, S. J., Mash, D. C., & Tyndale, R. F. (2012). The neuroprotective enzyme CYP2D6 increases in the brain with age and is lower in Parkinson's disease patients. *Neurobiology of Aging*, *33*(9), 2160–2171. http://doi.org/10.1016/j.neurobiolaging.2011.08.014
McClellan, A. J., Xia, Y., Deutschbauer, A. M., Davis, R. W., Gerstein, M., & Frydman, J. (2007). Diverse Cellular Functions of the Hsp90 Molecular Chaperone Uncovered Using Systems Approaches. *Cell*, *131*(1), 121–135. http://doi.org/10.1016/j.cell.2007.07.036
McDonald, P., Maizi, B. M., & Arking, R. (2013). Chemical regulation of mid- and late-life longevities in Drosophila. *Experimental Gerontology*, *48*(2), 240–249. http://doi.org/10.1016/j.exger.2012.09.006
Miyake, Y., Keusch, J. J., Wang, L., Saito, M., Hess, D., Wang, X., … Matthias, P. (2016). Structural insights into HDAC6 tubulin deacetylation and its selective inhibition. *Nature Chemical Biology*, *12*(9), 748–754. http://doi.org/10.1038/nchembio.2140
Mukherjee, S., Date, A., Patravale, V., Korting, H. C., Roeder, A., & Weindl, G. (2006). Retinoids in the treatment of skin aging: an overview of clinical efficacy and safety. *Clinical Interventions in Aging*. Dove Press. http://doi.org/10.2147/ciia.2006.1.4.327
Rissman, R. A., De Blas, A. L., & Armstrong, D. M. (2007, November). GABAA receptors in aging and Alzheimer's disease. *Journal of Neurochemistry*. http://doi.org/10.1111/j.1471-4159.2007.04832.x
Schuetz, A., Min, J., Allali-Hassani, A., Schapira, M., Shuen, M., Loppnau, P., … Arrowsmith, C. H. (2008). Human HDAC7 harbors a class IIa histone deacetylase-specific zinc binding motif and cryptic deacetylase activity. *Journal of Biological Chemistry*, *283*(17), 11355–11363. http://doi.org/10.1074/jbc.M707362200
Shamanna, R. A., Lu, H., Croteau, D. L., Arora, A., Agarwal, D., Ball, G., … Bohr, V. A. (2016). Camptothecin targets WRN protein: mechanism and relevance in clinical breast cancer. *Oncotarget*, *7*(12), 13269–13284. http://doi.org/10.18632/oncotarget.7906
Somoza, J. R., Skene, R. J., Katz, B. A., Mol, C., Ho, J. D., Jennings, A. J., … Tari, L. W. (2004). Structural snapshots of human HDAC8 provide insights into the class I histone deacetylases. *Structure*, *12*(7), 1325–1334. http://doi.org/10.1016/j.str.2004.04.012
Staker, B. L., Feese, M. D., Cushman, M., Pommier, Y., Zembower, D., Stewart, L., & Burgin, A. B. (2005). Structures of three classes of anticancer agents bound to the human topoisomerase I-DNA covalent complex. *Journal of Medicinal Chemistry*, *48*(7), 2336–2345. http://doi.org/10.1021/jm049146p
Stebbins, C. E., Russo, A. A., Schneider, C., Rosen, N., Hartl, F. U., & Pavletich, N. P. (1997). Crystal Structure of an Hsp90–Geldanamycin Complex: Targeting of a Protein Chaperone by an Antitumor Agent. *Cell*, *89*(2), 239–250. http://doi.org/10.1016/S0092-8674(00)80203-2
Strobl, G. R., von Kruedener, S., Stockigt, J., Guengerich, F. P., & Wolff, T. (1993). Development of a pharmacophore for inhibition of human liver cytochrome P-450 2D6: molecular modeling and inhibition studies. *J Med Chem*, *36*(9), 1136–1145. Retrieved from http://www.ncbi.nlm.nih.gov/pubmed/8487254
Tacutu, R., Thornton, D., Johnson, E., Budovsky, A., Barardo, D., Craig, T., … de Magalhaes, J. P. (2017). Human Ageing Genomic Resources: 2018 Update. *Doi.org*, 193326. http://doi.org/10.1101/193326
Takauji, Y., Wada, T., Takeda, A., Kudo, I., Miki, K., Fujii, M., & Ayusawa, D. (2016). Restriction of protein synthesis abolishes senescence features at cellular and organismal levels. *Scientific Reports*, *6*(1), 18722. http://doi.org/10.1038/srep18722
Vorinostat. (n.d.). Retrieved October 30, 2017, from https://www.drugbank.ca/drugs/DB02546
Walker, E. H., Pacold, M. E., Perisic, O., Stephens, L., Hawkins, P. T., Wymann, M. P., & Williams, R. L. (2000). Structural Determinants of Phosphoinositide 3-Kinase Inhibition by Wortmannin, LY294002, Quercetin, Myricetin, and Staurosporine. *Molecular Cell*, *6*(4), 909–919. http://doi.org/10.1016/S1097-2765(05)00089-4
Yang, J., Shamji, A., Matchacheep, S., & Schreiber, S. L. (2007). Identification of a Small-Molecule Inhibitor of Class Ia PI3Ks with Cell-Based Screening. *Chemistry and Biology*, *14*(4), 371–377. http://doi.org/10.1016/j.chembiol.2007.02.004
Ye, X., Linton, J. M., Schork, N. J., Buck, L. B., & Petrascheck, M. (2014). A pharmacological network for lifespan extension in Caenorhabditis elegans. *Aging Cell*, *13*(2), 206–215. http://doi.org/10.1111/acel.12163
Zhao, X., Zhang, Y., Strong, R., Grotta, J. C., & Aronowski, J. (2006). 15d-Prostaglandin J2 activates

peroxisome proliferator-activated receptor-gamma, promotes expression of catalase, and reduces inflammation, behavioral dysfunction, and neuronal loss after intracerebral hemorrhage in rats. *Journal of Cerebral Blood Flow and Metabolism : Official Journal of the International Society of Cerebral Blood Flow and Metabolism*, *26*(6), 811–820. http://doi.org/10.1038/sj.jcbfm.9600233

Zunino, F., & Capranico, G. (1990). DNA topoisomerase II as the primary target of anti-tumor anthracyclines. *Anti-Cancer Drug Design*, *5*(4), 307–17. Retrieved from http://www.ncbi.nlm.nih.gov/pubmed/1963303
